# Regulation of Enteroendocrine Cell Networks by the Major Human Gut Symbiont *Bacteroides thetaiotaomicron*

**DOI:** 10.3389/fmicb.2020.575595

**Published:** 2020-11-06

**Authors:** Amisha Modasia, Aimee Parker, Emily Jones, Regis Stentz, Arlaine Brion, Andrew Goldson, Marianne Defernez, Tom Wileman, L. Ashley Blackshaw, Simon R. Carding

**Affiliations:** ^1^Gut Microbes and Health Research Programme, Quadram Institute Bioscience, Norwich, United Kingdom; ^2^Core Science Resources, Quadram Institute Bioscience, Norwich, United Kingdom; ^3^Norwich Medical School, Faculty of Medicine and Health Sciences, University of East Anglia, Norwich, United Kingdom

**Keywords:** intestinal microbiota, *Bacteroides thetaiotaomicron*, germfree mice, enteroendocrine cells, short chain fatty acids

## Abstract

Gut microbes have critical roles in maintaining host physiology, but their effects on epithelial chemosensory enteroendocrine cells (EEC) remain unclear. We investigated the role that the ubiquitous commensal gut bacterium *Bacteriodes thetaiotaomicron* (Bt) and its major fermentation products, acetate, propionate, and succinate (APS) have in shaping EEC networks in the murine gastrointestinal tract (GIT). The distribution and numbers of EEC populations were assessed in tissues along the GIT by fluorescent immunohistochemistry in specific pathogen free (SPF), germfree (GF) mice, GF mice conventionalized by Bt or *Lactobacillus reuteri* (Lr), and GF mice administered APS. In parallel, we also assessed the suitability of using intestinal crypt-derived epithelial monolayer cultures for these studies. GF mice up-regulated their EEC network, in terms of a general EEC marker chromogranin A (ChrA) expression, numbers of serotonin-producing enterochromaffin cells, and both hormone-producing K- and L-cells, with a corresponding increase in serum glucagon-like peptide-1 (GLP-1) levels. Bt conventionalization restored EEC numbers to levels in SPF mice with regional specificity; the effects on ChrA and L-cells were mainly in the small intestine, the effects on K-cells and EC cells were most apparent in the colon. By contrast, Lr did not restore EEC networks in conventionalized GF mice. Analysis of secretory epithelial cell monolayer cultures from whole small intestine showed that intestinal monolayers are variable and with the possible exclusion of GIP expressing cells, did not accurately reflect the EEC cell makeup seen *in vivo*. Regarding the mechanism of action of Bt on EECs, colonization of GF mice with Bt led to the production and accumulation of acetate, propionate and succinate (APS) in the caecum and colon, which when administered at physiological concentrations to GF mice via their drinking water for 10 days mimicked to a large extent the effects of Bt in GF mice. After withdrawal of APS, the changes in some EEC were maintained and, in some cases, were greater than during APS treatment. This data provides evidence of microbiota influences on regulating EEC networks in different regions of the GIT, with a single microbe, Bt, recapitulating its role in a process that may be dependent upon its fermentation products.

## Introduction

A mutualistic relationship exists between the intestinal microbiota and the host in which commensal microbes provide the host with essential protective and metabolic functions, including fermentation of complex plant-based carbohydrates to produce metabolites such as short chain fatty acids (SCFAs) that are an important energy source for host cells (McNeil, 1984; Bergman, 1990). In turn, the host provides the microbiota with nutrients essential for their colonization and survival (Savage, 1977; Tremaroli and Backhed, 2012). Chronic metabolic diseases including metabolic syndrome, obesity and diabetes have been associated with structural and/or functional changes in the intestinal microbiota and principally, the prokaryome (Carding et al., 2015). The causal nature of these associations remains to be determined, although alterations in energy extraction from food (Backhed et al., 2004; Turnbaugh et al., 2006), increased nutrient harvesting (Tremaroli and Backhed, 2012) and appetite signaling (Plovier and Cani, 2017; Covasa et al., 2019) are potential mechanisms. Animal models and in particular germfree (GF) rodents have been instrumental in advancing our understanding of the complexity of the intestinal microbiota and providing mechanistic insights of microbial-host interactions at the epithelial interface (Tremaroli and Backhed, 2012).

Enteroendocrine cells (EECs) are scattered throughout the entirety of the epithelium of the gastrointestinal tract (GIT) and are key sensors of microbial metabolites in the intestinal lumen. They sense changes within the luminal environment and relay signals via the production and secretion of peptide hormones, which act on local nerve endings of the enteric nervous system or other cells within the intestinal mucosa that converge on hypothalamic feeding circuits to regulate and coordinate metabolism and food intake (Beutler et al., 2017). Via the circulatory system and vagal nerves their influence can extend beyond the GIT, affecting the function of organs such as the brain, liver and adipose tissues (Gribble and Reimann, 2016). EECs are divided into subgroups depending on their secreted hormones and location along the GIT. Prominent subsets include L, K and enterochromaffin cells (EC). L cells secrete mainly glucagon-like peptide-1 (GLP-1) or peptide YY and are found throughout the GIT but are more densely populated in the colon. K cells secrete glucose-dependent insulinotropic polypeptides (gastric inhibitory peptide, GIP) and are mainly found in the upper small intestine. Enterochromaffin cells, found throughout the GIT, make up the single largest population of endocrine cells in the intestinal epithelium and produce mainly serotonin or 5-hydroxytryptamine (5-HT) (Sjolund et al., 1983).

GLP-1 and GIP are the primary incretin hormones that cause the release of insulin from pancreatic beta-cells following meal ingestion (Baggio and Drucker, 2007). The administration of probiotic bacteria to obese and diabetic mice increases glucose tolerance, L cell number, intestinal proglucagon mRNA and plasma GLP-1 levels, suggesting that intestinal microbes can play a role in altering glucose homeostasis and EEC activity (Cani et al., 2008). However, our understanding of how the intestinal microbiota initiates signaling in EEC is incomplete. Key insights have been obtained from studies using wildtype or transgenic strains of germfree animals and by examining the impact of substrates and metabolites of microbiota metabolism. Evidence for the ability of the intestinal microbiota to influence L cells directly has come from germfree mice expressing a proglucagon reporter gene in which conventionalization with an unfractionated microbiota has been shown to modulate the L cell transcriptome in the ileum (Arora et al., 2018). Amongst microbial metabolites, SCFAs have been the most intensively studied with those produced from the fermentation of dietary fiber increasing GLP-1 and peptide YY (PYY) levels in tissues and plasma (Keenan et al., 2006; Zhou et al., 2006, 2008). SCFAs signal through G-protein coupled receptors (GPCRs) that co-localize with EECs (Karaki et al., 2006), such as free fatty acid receptor (FFAR) 1 and 3 (also known as GPR41 and 43, respectively, expressed on L cells) (Tazoe et al., 2008, 2009; Tolhurst et al., 2012; Nohr et al., 2013). GPR43-deficient mice display lower GLP-1 plasma levels and reduced glucose tolerance highlighting the importance of these receptors in microbial signaling in EECs (Tolhurst et al., 2012). In addition, oligofructose supplementation increases GIP plasma levels as well as affecting microbial composition (Girard, 2008; Tolhurst et al., 2012). Furthermore, GIP is involved in fat metabolism (Yip and Wolfe, 2000) and therefore is a potential target for microbiota modulation in obesity. Using two bacteria to conventionalize germfree mice a role for the GPR41 receptor in regulating host energy balance has been identified in a process involving bacterial modulation of PYY expression (Samuel et al., 2008). Aside from metabolic processes, the intestinal microbiota can affect neuronal signaling processes by altering 5-HT production. For example, the numbers of EECs are reduced in number in GF rats (Uribe et al., 1994), while the presence of indigenous spore-forming bacteria, mainly from the *Clostridial* spp., promotes 5-HT biosynthesis through increasing Tph1 expression, a rate-limiting enzyme involved in the biosynthesis of 5-HT tryptophan (Yano et al., 2015; Zelkas et al., 2015). Conversely, 5-HT stimulates the growth in culture of bacterial species including *E. coli* and *Rhodospirillum* (Oleskin et al., 1998), suggesting a bi-directional relationship exists between EEC signaling and the gut microbiota.

Collectively, these studies suggest the intestinal microbiota has profound effects on EECs including regulation of production and secretion of their peptide hormones, which may occur via products of microbial metabolism acting directly on EECs. Here, we have undertaken a study to obtain more evidence of these putative mechanisms using conventional and GF mice to investigate the role that the ubiquitous and prominent commensal gut bacterium *Bacteroides thetaiotaomicron* (Bt) and its major metabolic output (acetate, propionate and succinate) (Hooper et al., 2002; Wrzosek et al., 2013; Curtis et al., 2014) have on EEC networks in the murine GIT.

## Materials and Methods

### Bacterial Strains and Culturing

Bt (VPI 5482, ATCC) was grown anaerobically at 37°C in brain heart infusion medium (Oxoid) supplemented with 15 μM hemin. *Lactobacillus reuteri* (Lr; 100-23, DSMZ) was grown anaerobically at 37°C in MRS medium (Difco Laboratories).

### Animal Handling

C57BL/6 mice of 8–12 weeks of age were housed in a specific pathogen free (SPF) Disease Modeling Unit (DMU) at the University of East Anglia (UEA), Norwich, United Kingdom and were maintained on standard chow at all times throughout the study. All experiments were conducted in accordance with the Home Office Animals (Scientific Procedures) Act 1986 under the license number PPL80/2545 at the UEA. C57BL/6 germfree (GF) mice were maintained in sterile isolators in the Quadram Institute Germ Free Facility within the DMU with the GF status being continuously monitored by microscopy, aerobic and anaerobic culturing, and PCR for bacterial contamination. GF mice were conventionalized by administrating 0.1 ml (1.4 × 10^9^ cells/ml) of Bt or Lr in sterile PBS by oral gavage. Conventionalized GF mice were maintained in individual ventilated cages for up to 10 days. To assess extent of colonization, contents of the GIT were cultured under anaerobic conditions and colony counts determined. Additional aerobic and anaerobic cultures were performed to exclude contamination. In some experiments GF mice were administered via their drinking water a cocktail of APS at levels comparable to those in the gut lumen consisting of sodium acetate (95 μM Sigma-Aldrich), sodium propionate (29 μM, Sigma-Aldrich) and sodium succinate (5.6 μM, Sigma-Aldrich) (Mineo et al., 2006) for 10 days after which the APS-containing drinking water was replaced with regular drinking water (wash out) for a further 10 days.

### Blood and Tissue Sampling

Sampling was carried out at the same time of day for all experiments. Blood samples were taken by cardiac puncture following euthanasia with 0.1 ml of Dipeptidyl peptidase IV (DPP-IV) inhibitor (BIO-TECHNE LTD.) per ml of blood, centrifuged at 1,000–2,000 × *g* for 10 min and the serum removed, aliquoted and stored at −20°C prior to analysis. The entire intestinal tract was excised, the contents removed by flushing with sterile Dulbecco’s Phosphate Buffered Saline (DPBS), prior to dividing into anatomically distinct segments (duodenum, jejunum, ileum, proximal colon and distal colon) that were fixed in 10% neutral buffered formalin (Sigma-Aldrich) for 24 h at 20–22°C followed by 24 h in 70% ethanol at 4°C. Tissues were then processed through a xylene/alcohol dehydration and clearing series followed by wax infiltration. Segments were embedded in paraffin wax prior to sectioning (5 μm) and mounting on SuperFrost^®^ Plus glass slides (VWR). ELISA (Millipore, MMHMAG-44K) was used to quantitate GLP-1 levels in serum.

### Intestinal cfu

Content was obtained from all regions of the intestine (duodenum, jejunum, ileum, cecum, proximal colon and distal colon) from GF mice 5 days post- conventionalization with Bt, and weighed, prior to addition of 400 μl PBS to each sample. Samples were briefly vortexed, centrifuged at 1000 rpm for 10 min at 20–22°C and serial two-fold dilutions carried out and plated on BHI agar plates. Plates were incubated in an anaerobic cabinet (37°C, 5% CO_2_) for 48 h. Colonies were counted and used to calculate the CFU/g of contents at 5-days post-colonization with Bt in germ-free mice.

### Immunohistochemistry

Tissue sections were rehydrated through histoclear and a graded ethanol series. Following washing in dH_2_0, slides were heated in citric acid buffer (10 mM, pH 6) (Sigma-Aldrich) for antigen retrieval, washed further in Tris-buffered saline with Tween-20 (TBS-T) and incubated for 16 h at 4°C with either a rabbit polyclonal anti-GLP-1 (ab22625, Abcam), mouse monoclonal anti-GLP-1 antibody (ab23468, Abcam), rabbit monoclonal anti-GIP antibody (ab209792, Abcam), mouse monoclonal anti-GIP (021-04, Santa Cruz), rabbit polyclonal anti-Chromogranin A (ChrA) antibody (sc-13090, Santa Cruz Biotechnology), goat polyclonal anti-5-HT antibody (ab66047, Abcam) and Hoechst nuclear stain (Thermo Fisher). Unless specified, control antibodies were obtained from Abcam; rabbit IgG (ab37415) and monoclonal IgG (ab172730), mouse IgG2a (ab18415), mouse IgG_1_ (IS5-21F5, Miltenyi Biotech), and goat IgG (ab37373). Tissues were washed in TBS-T and incubated with Alexa Fluor594 goat anti-rabbit Ig (27117, Invitrogen), Alexa Flour488 anti-mouse IgG (Thermo Fisher A11001), or Alexa Fluor594 donkey anti-goat Ig (A11057, Invitrogen) for 30 min at 20–22°C. Tissues were mounted using ProLong^TM^ Diamond Antifade mountant (Thermo Fisher). The hemi-villous crypt region in each section of the GIT was used to enumerate EECs (Supplementary Figure 1) with the total number of epithelial cells in the same hemi-villous crypts also determined. A minimum of 20 hemi-villus crypts were counted for each section with at least 10 sections from each tissue sample and experimental group using a minimum of 3 mice per group to obtain EEC cell counts.

### Metabolite Analysis by Nuclear Magnetic Resonance (NMR)

Acetate, butyrate, propionate and succinate were quantified in the contents of the duodenum, distal colon (SPF, GF, and GF-Bt), and cecum (SPF, GF, GF Bt, and GF Lr) (*n* = 5 ea.) using ^1^H NMR spectroscopy. Samples were prepared by mixing ∼50 mg of the sample with 12 times the volume of phosphate buffer-D2O (0.1 M K_2_HPO_4_, 0.1 M NaH_2_PO_4_, 145.1 μM TSP-d4 mixed 1:1 with deuterium oxide [D2O]). The ^1^H NMR spectra were recorded on a 600 MHz Bruker Advance spectrometer (Bruker BioSpin GmbH, Germany). Each ^1^H NMR spectrum was acquired with 64 scans, a spectral width of 12,500 Hz, and an acquisition time of 2.62 s. The “noesygppr1d” pre-saturation sequence was used to suppress the residual water signal with low power selective irradiation at the water frequency during the recycle delay (D1 = 2 s) and mixing time (D8 = 0.15 s). A 90_ pulse length of 8.8 μs was set for all samples. Spectra were transformed with a 0.1 Hz line broadening and manually phased in TopSpin 3.9.1, and the chemical shift scale referenced to TSP. The spectra were then baseline corrected, removing the broad envelope between 0.7 and 4.5 ppm using AMIX 3.9.15 (underground removal tool, filter width = 20 Hz). Acetate, butyrate, propionate, and succinate were quantified using the Chenomx NMR Suite 8.12.

### Intestinal Crypt Isolation and Culture

The intact small intestine was flushed with ice-cold DPBS, opened longitudinally and villi removed by gentle scraping using a glass coverslip. Tissues were then cut into 5–8 mm pieces, vigorously washed 5 times in ice-cold DPBS and transferred to 50 ml tubes containing 15 ml Gentle Cell Dissociation Reagent (Stem Cell Technologies) and incubated at 20–22°C for 15 min on a rolling platform. Tissues were then washed in ice-cold DPBS to release the crypts and filtered to remove excess debris using a 70 μm cell strainer (Corning). The crypt suspensions were then centrifuged at 300 × *g* for 3 min at 20–22°C. Supernatant was removed, and crypt pellets were resuspended in IntestiCult Organoid Growth Medium Mouse (Stem Cell Technologies) supplemented with Penicillin/Streptomycin, and Y-27632 ROCK inhibitor (Stem Cell Technologies) to prevent anoikis. The crypts were then plated onto glass coverslips (Agar Scientific Ltd.) in 24 well cell culture plates (Greiner Bio-One Ltd.) coated with 1:20 dilution (in DPBS) of Matrigel Basement Membrane Matrix (Scientific Laboratory Supplies) for 20–24 h to form semi-confluent monolayers. Monolayers were fixed in 10% neutral buffered formalin, washed with DPBS and permeabilized with 0.25% Triton X100 in DPBS, then incubated in blocking buffer (DPBS containing 10% goat serum (Sigma-Aldrich). Cultures were then incubated with rabbit polyclonal anti-GLP-1 antibody (ab22625, Abcam), rabbit monoclonal anti-GIP antibody (ab209792, Abcam), or rabbit polyclonal anti-ChrA antibody (sc-13090, Santa Cruz Biotechnology) and mouse anti-E-cadherin antibody (610181 BD Transduction Laboratories) for 2 h at 20–22°C. Following further washes in DPBS, monolayers were incubated for 30 min with the secondary antibodies, Alexa Fluor594 goat anti-rabbit (37117, Invitrogen) and Alexa Fluor488 goat anti-mouse (A11001, Invitrogen), followed by Hoescht (Thermo Fisher). The monolayers were washed with H_2_0, coverslips carefully removed and mounted on glass slides using ProLong Diamond Antifade mountant (Thermo Fisher).

### Statistical Analysis

For box and whisker plots, the box extends from the 25th to 75th percentiles with the horizontal line within the box representing the median and the whiskers representing the minimum and maximum values. Other graphed date sets are expressed as mean ± SEM. For immunohistochemistry, data was analyzed by fitting a mixed model (GraphPad Prism 8.0). Statistical analysis was performed using a two-way ANOVA with *p* < 0.05 defined as significant with Geisser-Greenhouse correction not used.

## Results

To determine the impact of the intestinal microbiota on EEC networks, the distribution and number of several key EEC subsets (i.e., K, L, and EC cells) were compared in SPF and GF mice, and in GF mice after mono-conventionalization with the commensal gut bacteria Bt or Lr, or after administering physiological concentrations of the major fermentation products (acetate, propionate, and succinate; APS) of Bt. EEC numbers were determined by counting antibody stained cells within the hemi-villous crypt region (Supplementary Figure 1) counting a minimum of 20 hemi-villus crypts for each section and at least 10 sections from each tissue sample and experimental group using a minimum of 3 mice per group.

### Distribution of EECs in the GIT of SPF and GF Mice

**FIGURE 1:**
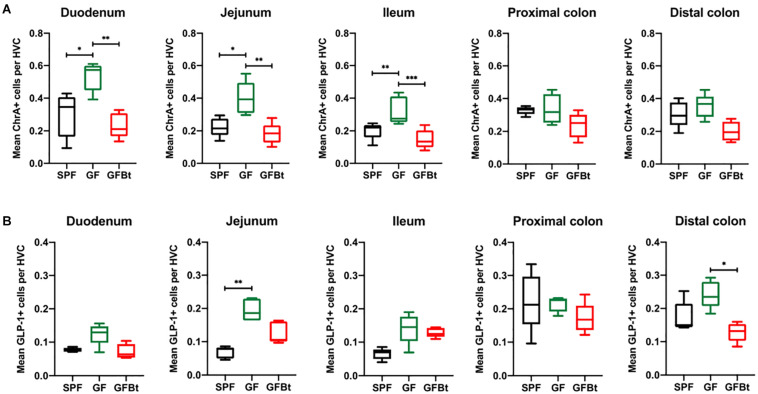
Impact of colonization of GF mice with Bt on the distribution of ChrA^+^ and GLP-1^+^ EECs. ChrA^+^ **(A)** and GLP-1^+^ **(B)** cells were counted in the hemi-villus crypt (HVC) of sections of tissue obtained from different regions of the GIT of SPF (*n=10*), GF (*n=11*) and GF mice mono-colonized by Bt (GFBt, *n=10*) mice. The box of the box whisker plots extends from the 25th to 75th percentiles with the horizontal line within the box representing the median and the whiskers representing the minimum and maximum values. ^∗^*p* < 0.05, ^∗∗^*p* < 0.01, ^***^p < 0.001.

The small intestine of adult SPF and GF mice was divided into the anatomically distinct duodenum, jejunum and ileum. Although EECs can express more than one hormone (Egerod et al., 2012; Habib et al., 2012; Sykaras et al., 2014), they can be globally identified by ChrA expression which is stored and secreted by the majority of EECs (Massironi et al., 2016). Immunohistochemical staining of formalin fixed sections showed that cells reactive with anti-ChrA antibodies had the funnel shape morphology typical of EECs and were evenly distributed along the length of the intestine of SPF mice (Figure 1A and Supplementary Figure 2). Non-specific, background staining with isotype matched control antibodies was very low or absent (Supplementary Figure 2). In GF mice all regions of the small intestine contained significantly higher numbers (*p* < 0.05 to 0.01) of ChrA-expressing EECs compared to SPF mice (Figure 1A). In the colon, the distribution and number of ChrA-expressing cells were similar in SPF and GF mice with no regional differences being apparent after comparing the proximal and distal colon (Figure 1A). To assess if the differences and similarities noted in ChrA^+^ EECs in SPF and GF mice reflected those of distinct populations of EECs, the distribution and number of GLP-1, GIP and 5-HT expressing cells were examined.

### Regional Variation in Individual EEC Populations in the GIT of SPF and GF Mice

**FIGURE 2:**
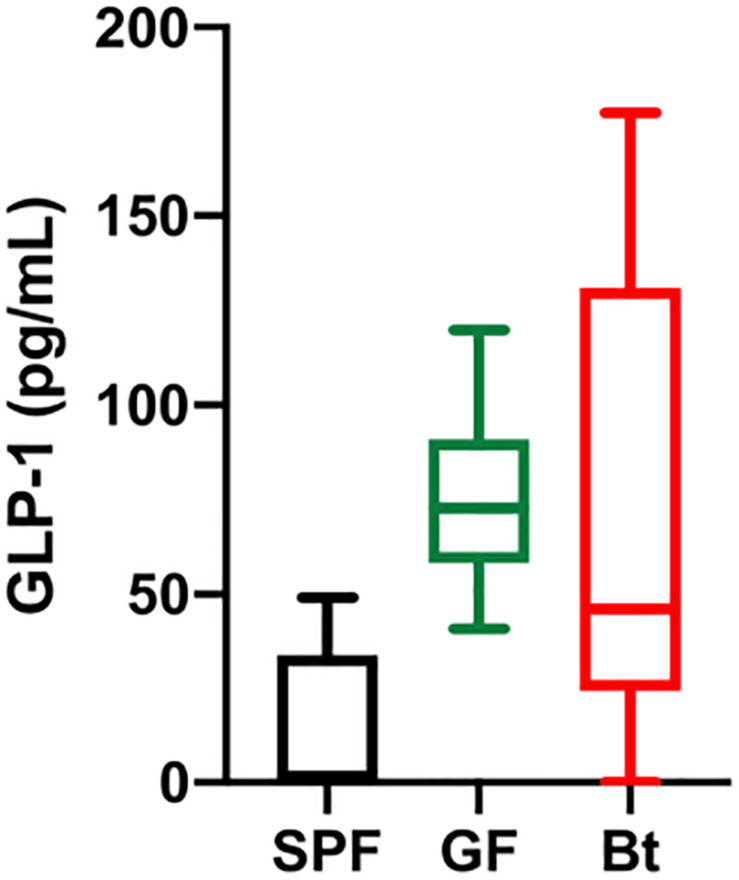
Serum GLP-1 levels. GLP-1 levels in the serum of SPF (*n=5*), GF (GF, *n=11*) and Bt mono-colonized GF mice (GFBt, *n=15*) were determined by ELISA. The box of the box whisker plots extends from the 25th to 75th percentiles with the horizontal line within the box representing the median and the whiskers representing the minimum and maximum values.

**FIGURE 3:**
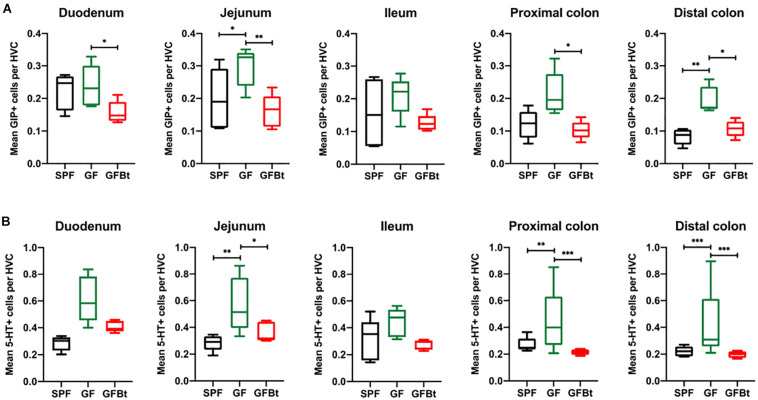
Impact of colonization of GF mice with Bt on the distribution of GIP^+^ and 5-HT^+^ EECs. GIP^+^ **(A)** and 5-HT^+^ **(B)** cells were counted in the hemi-villus crypt (HVC) in sections of tissue obtained from different regions of the GIT of SPF (*n=10*), GF (*n=11*) and GF mice mono-colonized by Bt (GFBt, *n=10*) mice. The box of the box whisker plots extends from the 25th to 75th percentiles with the horizontal line within the box representing the median and the whiskers representing the minimum and maximum values. ^∗^*p* < 0.05, ^∗∗^*p* < 0.01, ^*⁣**^p < 0.001.

Analysis of GLP-1^+^ cells in GF and SPF mice using two different (mouse and rabbit-derived) anti-GLP-1 antibodies revealed that GLP-1^+^ cells were equivalent along the entire length of the small intestine in both groups of animals with the exception of the jejunum where they were present in significantly higher numbers (*p* < 0.01) in GF mice (Figure 1B and Supplementary Figures 2 and 4). Analysis of plasma levels of GLP-1 in fed mice showed that GF mice had higher levels than SPF mice, although the differences were not statistically significant (Figure 2). The distribution of GIP-expressing EECs in the small intestine of SPF and GF animals was the same as that of GLP-1 expressing EECs with significant differences only evident in the jejunum (Figure 3A and Supplementary Figure 3). In the colon, and in contrast to GLP-1 expressing EECs that were similar in SPF and GF animals, an approximately two-fold increase in GIP-1 expressing cells were seen in the distal colon of GF mice (0.188 ± 0.02 versus 0.084 ± 0.014 cells/hemi-villus crypt of GF and SPF mice, respectively; Figure 3A). In light of previous inconsistent findings regarding the presence of GIP-expressing EECs in the colon (Jorsal et al., 2017; Billing et al., 2019; Roberts et al., 2019), we used two different anti-GIP (mouse and rabbit-derived) antibodies to identify GIP expressing cells in the colon of both SPF and germfree mice (Supplementary Figure 5). After Bt-conventionalization the levels of GIP expressing cells along the GIT were reduced to the levels seen in SPF mice (Figure 3A). 5-HT expressing EEC also displayed region-specific difference in their distribution in the GIT of GF versus SPF mice (Figure 3B and Supplementary Figure 3) with significantly higher numbers of positive cells (*p* < 0.01) in the jejunum and throughout the colon of GF mice (Figure 3B). It was not possible to detect any discrete staining with isotype matched control antibodies (Supplementary Figure 3). The regional variations in EEC subset numbers is unlikely to be due to any bias in the sectioning or presentation of the tissues as the number of epithelial cells within the hemi-villous crypts of sections of the same regions of the GIT from different mouse strains were equivalent (Supplementary Figure 1).

### EEC in Cultured Intestinal Crypt-Derived Epithelial Monolayers

The ability to establish cultures of the intestinal epithelium that reflect the architecture and distribution of differentiated cell types seen *in vivo* (Sato et al., 2009; Sato and Clevers, 2013) provides a valuable and tractable *in vitro* system to interrogate microbe-host cell interactions at the molecular and cellular level. To determine if such culture systems can faithfully replicate the different profiles of EECs seen in sections of preserved tissues of SPF and GF mice, we examined EEC in monolayer cultures established from small intestinal crypts of SPF, GF, Bt conventionalized GF mice, and GF treated with APS.

Representatives of the differentiated epithelial cell lineages including EEC and mucus-producing goblet cells were readily detected in cultured two-dimensional epithelial cell monolayers (Supplementary Figure 6). However, analysis of EEC populations in the cultured monolayers revealed inconsistencies in the distribution of EEC subsets compared to tissues preserved and processed directly *ex vivo*. In particular, the epithelial monolayer cultures established from GF mice showed no differences in the number of GLP-1^+^ or ChrA^+^ cells compared to those from SPF mice. By contrast, a significant increase in GIP^+^ cells (*p* = 0.005) was seen in monolayer cultures established from GF mice compared to SPF mice small intestine (Supplementary Figure 6b), similar to that seen in intact tissue sections (Figure 3A). Generally, therefore, our analysis of secretory cell cultures from whole small intestine showed that intestinal monolayers are variable and with the possible exclusion of GIP expressing cells, do not accurately reflect the EEC cell makeup seen *in vivo*. We therefore relied on immunohistochemistry of intact tissues for the analysis and more accurate enumeration of EECs in subsequent experiments.

### Bt Mono-Conventionalization of GF Mice Alters EEC Populations *in vivo*

The ability of commensal gut microbes to directly influence the makeup of the EECs network was assessed by conventionalizing GF mice with Bt, a universal and prominent member of the mammalian intestinal microbiota (Salyers, 1984) and comparing the distribution and numbers of EECs populations in the small and large intestine pre- and 10 days post-conventionalization. Bt was found throughout the length of the GIT of conventionalized mice which individually showed considerable variation in colonizing density based on cfu determinations of the luminal contents of different regions of the GIT (Supplementary Table 1). However, a consistent finding in all conventionalized animals was that the cecum and colon contained the highest levels of Bt as previously noted (Wrzosek et al., 2013; Curtis et al., 2014), and Bt colonization resulted in significant changes in EEC populations to the extent that they more closely resembled the profile and number of EEC seen in SPF mice. This was exemplified by the analysis of ChrA^+^ EECs pre- and post-conventionalization which showed a significant reduction in ChrA^+^ EECs throughout the small intestine of Bt conventionalized mice to levels comparable to that of SPF mice (*p* < 0.01to 0.001, Figure 1A and Supplementary Table 2). In the colon, Bt colonization also reduced the number of ChrA-expressing cells compared to both SPF and GF mice although the differences were not statistically significant (Figure 1A and Supplementary Table 2).

Analysis of individual EEC populations revealed subtle differences in the impact of Bt colonization on their regional distribution and/or numbers. For GLP-1 expressing EECs the effect of Bt was most apparent in the distal colon where it significantly reduced (*p* < 0.05) the number of positive cells (Figure 1B and Supplementary Table 2). By contrast, the impact of Bt conventionalization on GIP^+^ EECs was more profound with significant reductions (*p* < 0.05 to 0.01) in cell numbers seen in both the small (duodenum and jejunum) and large (proximal and distal) intestine post-Bt conventionalization making them comparable to that of SPF mice (Figure 3A and Supplementary Table 2). A similar effect was noted for 5-HT-expressing EECs with significant reductions (*p* < 0.05) post-Bt conventionalization seen in the jejunum and in particular, throughout the colon (*p* < 0.001) (Figure 3B and Supplementary Table 2).

### The Effects of Bt on EEC Networks Are Not Seen With an Unrelated Gut Commensal Bacterium

**FIGURE 4:**
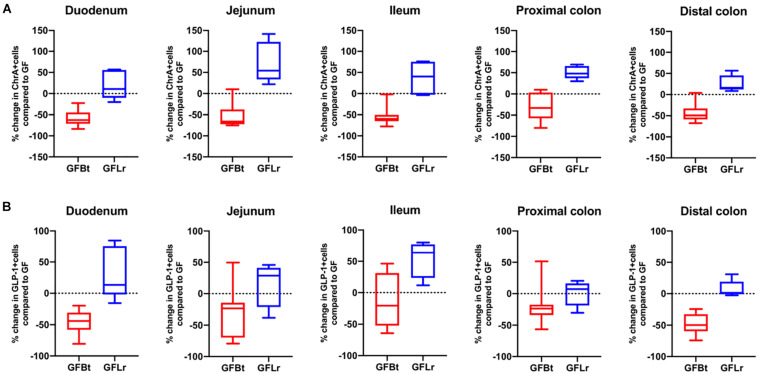
The effect of Bt on ChrA and GLP-1 EECs is not replicated with another gut commensal bacterium. **(A)** Percentage change of ChrA^+^ **(A)** or GLP-1^+^ **(B)** EECs throughout the GIT of GF mice after mono-colonization with Bt (GFBt, *n=10*) or Lactobaccillus reuteri (GFLr, *n=5*) compared to the number of GIP^+^ cells in GF (*n=11*) mice, which is shown as a dashed horizontal line and is set at zero. The box of the box whisker plots extends from the 25th to 75th percentiles with the horizontal line within the box representing the median and the whiskers representing the minimum and maximum values.

**FIGURE 5:**
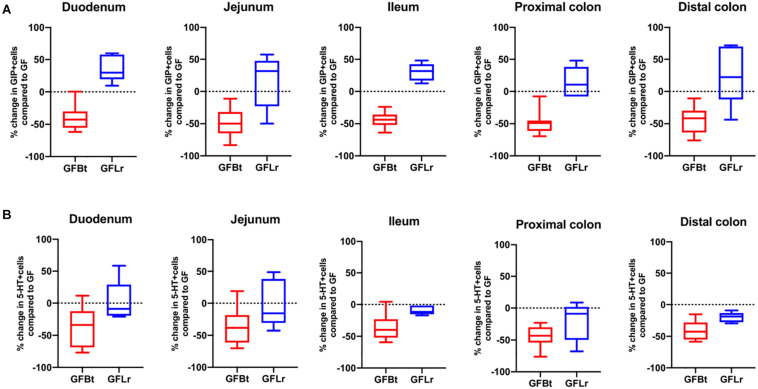
The effect of Bt on GIP and 5-HT EECs is not replicated with another gut commensal bacterium. **(A)** Percentage change of GIP^+^ **(A)** or 5-HT^+^ **(B)** EECs throughout the GIT of germfree mice after mono-colonization with Bt (GFBt, *n=10*) or Lactobaccillus reuteri (GFLr, *n=5*) compared to the number of GIP^+^ cells in GF (*n=11*) mice, which is shown as a dashed horizontal line and is set at zero. The box of the box whisker plots extends from the 25th to 75th percentiles with the horizontal line within the box representing the median and the whiskers representing the minimum and maximum values.

To determine if the effects of Bt on EEC networks after colonizing GF mice were specific to this bacterium, GF mice were conventionalized with a strain of Lr (100-23) isolated from the rat GIT that is able to stably colonize GF mice (Wesney and Tannock, 1979). The data shown in Figures 4, 5 (and Supplementary Table 3) show striking differences in the effects of Bt and Lr on EEC networks in the GIT post-conventionalization. Whereas Bt generally reduces the number of EECs in GF mice, Lr either had no significant effect (GLP-1^+^ cells, Figure 4B) or the opposite effect and significantly increased numbers of EECs as seen in the jejunum and proximal colon for ChrA^+^ cells (Figure 4A), in the duodenum for GIP^+^ cells (Figure 5A), and throughout the colon for 5-HT^+^ cells (Figure 5B).

### APS Reproduces the Effect of Bt Conventionalization on EECs Cells *in vivo*

Considering that EECs are highly enriched in free fatty acid receptors that contribute to physiological responses to microbially produced metabolites and SCFA (Lu et al., 2018), we investigated if these products, and in particular the fermentation products of Bt, mediated the effects of Bt on EEC networks.

First, we set out to confirm that the principle fermentation products of *Bacteroides* polysaccharide metabolism (acetate, propionate and succinate) (Hooper et al., 2002; Wrzosek et al., 2013; Curtis et al., 2014) were produced in Bt-conventionalized mice. NMR-based analysis was used to quantify these metabolites in the luminal contents of the duodenum, cecum and distal colon, 10 days post-conventionalization. As shown in Supplementary Table 4, all of the metabolites were present at very low levels (0.01–0.09 mM) in the GIT of GF mice. Post Bt colonization, the levels of acetate, succinate and propionate increased in the cecum and distal colon (0.26–2.35 mM). Strikingly, the levels of succinate in the cecum increased by >200-fold compared to those in both GF and SPF mice, as seen previously in C3H/HEJ mice post Bt colonization (Curtis et al., 2014). As expected, Bt conventionalization had no impact on butyrate levels (0.01 to 0.02 mM and 0.01 to 0.02 mM pre- and post-conventionalization, respectively) consistent with Bt not being a butyrate producer. To determine if the Bt fermentation products acetate, propionate and succinate (APS) could reproduce and provide an explanation for the effects of the bacterium itself on EEC populations seen *in vivo*. GF mice were administered via their drinking water APS in amounts corresponding to those present in the cecum of SPF rodents maintained on regular chow (95 μM acetate, 29 μM propionate and 5.6 μM succinate) (Mineo et al., 2006). Ten days later intestinal tissues were removed and examined for EECs.

**FIGURE 6:**
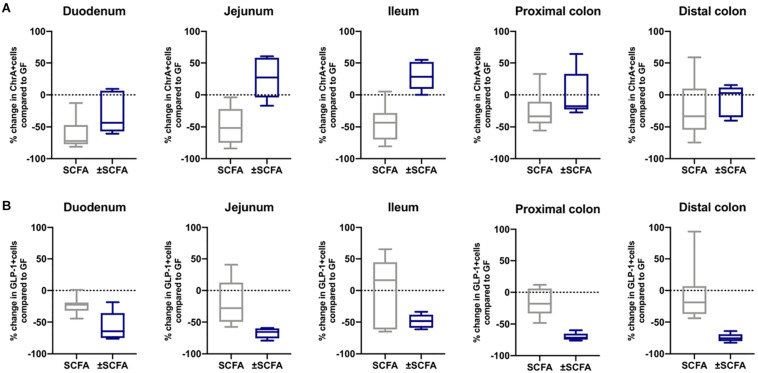
Effects of APS on ChrA^+^ and GLP1^+^ EECs in GF mice. ChrA^+^ **(A)** and GLP-1^+^ **(B)** EECs were enumerated in tissue sections obtained from different regions of the GIT of GF either 10 days after administering APS (95 μM acetate, 29 μM propionate and 5.6 μM succinate) via drinking water, or after an additional 10 day washout period (± APS) and were compared to the number of ChrA^+^ and GLP-1^+^ cells in GF mice (*n=11*), which is shown as a dashed horizontal line and is set at zero. The box of the box whisker plots extends from the 25th to 75th percentiles with the horizontal line within the box representing the median and the whiskers representing the minimum and maximum values. The horizontal dashed line represents values of GF mice which was set at zero.

**FIGURE 7:**
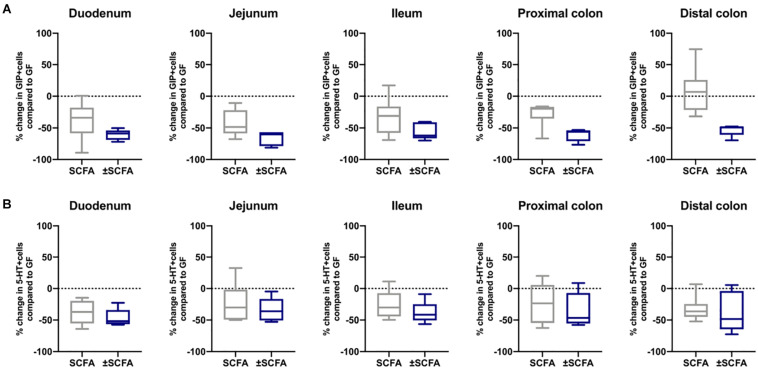
Effects of APS on GIP^+^ and 5-HT^+^ EECs in GF mice. GIP^+^ **(A)** and 5-HT^+^ **(B)** EECs were enumerated in tissue sections obtained from different regions of the GIT of GF either after 10 days after administering APS (95 μM acetate, 29 μM propionate and 5.6 μM succinate) via drinking water for 10 day, or after an additional 10 day washout period (±APS) and were compared to the number of GIP^+^ and 5-HT^+^ cells in GF mice (*n=11*), which is shown as a dashed horizontal line and is set at zero. The box of the box whisker plots extends from the 25th to 75th percentiles with the horizontal line within the box representing the median and the whiskers representing the minimum and maximum values.

The impact of Bt-APS on ChrA^+^ cells was comparable to that seen after Bt conventionalization of GF mice with a reduction in the number of positive cells throughout the GIT and in particular in the small intestine which showed an approximate 50% reduction (Figure 6A), as seen with Bt conventionalization (Figure 1). The impact of APS on GLP-1^+^ cells was more variable with the most apparent reductions in positive cells seen in the duodenum, jejunum and proximal colon (Figure 6B). Bt-APS administration had a similar effect on GIP+ (Figure 7A) and 5-HT^+^ cells (Figure 7B) as that of ChrA^+^ cells with reductions in positive cells seen throughout the small intestine and the colon.

To determine if the effects of administering Bt-APS on EEC were dependent upon constant exposure to APS, GF mice were treated with Bt-APS for 10 days followed by a 10 day wash out period prior to EEC analysis. For ChrA-expressing cells the removal of Bt-APS led to a rebound effect and increase in the number of positive cells in the small intestine and in particular, in the jejunum and ileum where the levels significantly exceeded that of non-treated GF mice (Figure 6A and Supplementary Table 5). Amongst individual EEC subsets some interesting and contrasting effects were noted. For GLP-1-expressing cells the removal of Bt-APS resulted in further and significant reductions in the proportion of positive cells throughout the small intestine and colon (Figure 6B and Supplementary Table 5) with the number of GLP-1 expressing cells in the small intestine now being comparable to that of SPF mice. A similar albeit more regionalized effect was seen for GIP-expressing cells with the withdrawal of Bt-APS resulting in further significant reductions in positive cells in the jejunum and in the proximal and distal colon (Figure 7A and Supplementary Table 5). In contrast, the withdrawal of Bt-APS had no significant impact on 5-HT expressing cells (Figure 7B and Supplementary Table 5).

## Discussion

A major challenge to developing a more detailed understanding of the nature of microbiota-EEC interactions that underpins the development of new evidence-based treatments for disorders affecting the GIT and other connected organ systems, is identifying which microbes are important and how they contribute to this crosstalk. Our recent study describing the ability of Bt to promote neurogenesis within the enteric nervous system of Bt conventionalized GF mice with accompanying effects on L-cells and EC cells (Aktar et al., 2020) prompted us to investigate in greater depth the mechanism and selectivity of the effect of Bt on EEC in the current study. The results presented here show for the first time that Bt is directly involved in shaping EEC networks throughout the mouse GIT in a process that is related to, and may be dependent on, their metabolism and production of succinate and the SCFAs acetate and propionate.

There are several cases reported in the literature of individual gut microbes, when administered exogenously, affecting host physiology. These include normalization of an autism phenotype seen in offspring of immunologically challenged dams by *B. fragilis* (Hsiao et al., 2013). Similarly, microbe-depleted mice showed altered behavior (Hoban et al., 2016) and probiotic treatment of normal mice with *L. rhamnosus* reduces anxiety and depression-related behaviors (Bravo et al., 2011). The SCFA receptor Gpr41 expressed on EEC, was shown, using GF mice, to act as a regulator of host energy balance through effects that are dependent upon the gut microbiota, although this was considered to be via cholecystokinin-containing cells of the upper intestine rather than L-cells. Such studies exemplify the ability of individual species to have potent influences at sites remote from the GIT. Their fundamental mechanisms of action, however, are not addressed. We reinstated one microbial species (Bt, a major constituent of the mammalian intestinal microbiota) in mice that were otherwise GF from birth. Thus, the role of Bt in postnatal development of EEC could be determined without quorum- or network-mediated effects that could be responsible in antibiotic-depleted or exogenously supplemented normal animals. In a similar study to ours, GF mice were mono-colonized with either *E. coli* or Bt for 4 weeks (Wichmann et al., 2013). This study reported that GLP-1 positive cells in the proximal colon are increased by colonization with Bt but not by *E. coli*, although there were regional differences to our results. This study also found serum GLP-1 increased in GF mice as we did. Other studies conventionalizing GF mice with specific microbiota agree with our findings. For example, Turnbaugh and colleagues (Turnbaugh et al., 2006) showed that conventionalization of GF mice with an obesity-associated mouse gut microbiome induces an increased capacity for energy harvest (Turnbaugh et al., 2006). Reigstad and co-workers (Reigstad et al., 2015), through the use of GF and humanized mice, showed that gut microbiota are important determinants of enteric 5-HT production and homeostasis, as we observed in the proximal and distal intestine of Bt conventionalized GF mice. We also demonstrated the selectivity of the effect of Bt on EEC networks as seen by the inability of another unrelated rodent gut commensal bacterium, Lr, to replicate the effects of Bt on EECs in mono-conventionalized GF mice.

The ability of Bt to influence EEC throughout the GIT is perhaps not surprising considering *Bacteroides* species are found in close association with the mucus that coats intestinal epithelial cells (Bry et al., 1996) and are therefore juxtaposed with EEC. It is important to note, however, that the impact of Bt on EEC is not uniform throughout the GIT with some but not all EEC subsets being modulated to the same degree, suggestive of a both a regionalized and subset specific effect of Bt on EEC. Unlike Billing et al. and Roberts et al. (Billing et al., 2019; Roberts et al., 2019) who using “omics based approaches were unable to detect *Gip* expression in the mouse colon, we detected the presence of GIP-expressing cells in the proximal and distal colon of both germfree and SPF mice.” Whilst we cannot entirely exclude the possibility that this is the result of non-specific antibody reactivity, similar findings to ours have been reported in human studies using immunohistochemistry and mRNA analyses to detect GIP expression in the distal colon (Jorsal et al., 2017). Discrepancies in detecting GIP expressing cells in the mouse colon may, in addition to experimental design and methodological differences, be related to variations in environmental conditions within different animal facilities. Each facility has their own unique combination of various and numerous attributes of animal husbandry that impact on the bacterial communities within each facility and on the microbiome of their occupants that can influence host physiology and phenotype (Rausch et al., 2016).

It is particularly noteworthy that Bt exerts effects on EEC in the small intestine, which is at odds with the conventional view of Bt being a resident of the anoxic cecum and colon. However, we have shown the ability of Bt to colonize both the small and large intestine of GF mice, comparable with its presence in regions of the small intestine of healthy humans (Mallory et al., 1973). This could therefore provide a possible route for its global effect on EEC networks. Alternatively, Bt might act via non-cognate interactions and through the production of metabolites or other mediators that are absorbed from the intestinal lumen and then disseminated throughout the body via the circulatory or nervous systems.

Among the various pathways and products that could be responsible for the effects of Bt on EEC we investigated their major products of polysaccharide fermentation, acetate, propionate and succinate (Wrzosek et al., 2013; Curtis et al., 2014). We confirmed that all three metabolites are produced and accumulate in the cecum and colon of Bt-conventionalized GF mice. High levels of succinate are particularly noteworthy and replicate prior studies of Bt colonized, antibiotic-pre-treated, C3H/HeJ mice demonstrating a 200-fold increase in cecal succinate levels post-Bt colonization (Curtis et al., 2014). Amongst SCFA, acetate, propionate, and butyrate are the most abundant (≥95%) (Cook and Sellin, 1998) and are present in an approximate molar ratio of 60:20:20 in the colon and stool (Cummings et al., 1987; Hijova and Chmelarova, 2007; Binder, 2010). The prominence within the human colon of *Bacteroides* which make up ∼25% of the total anaerobes (Salyers, 1984), and are adept glycan metabolisers (Salyers et al., 1977) and producers of high levels of acetate (Wrzosek et al., 2013; Curtis et al., 2014), helps explain the prominence of acetate amongst SCFA in the colonic lumen. The importance of acetate and other SCFAs to the host is exemplified by the fact that they provide ∼10% of our daily caloric requirements (McNeil, 1984; Bergman, 1990). In addition, propionate stimulates intestinal gluconeogenesis (De Vadder et al., 2014) and contributes to protecting the integrity of the blood-brain barrier (Hoyles et al., 2018), whereas succinate is a key intermediary in several metabolic pathways, playing an important role in the elimination of reactive oxygen species (Tretter et al., 2016). What emerged from our study is that a mixture of acetate, propionate and succinate administered in physiologically appropriate concentrations and molar ratios (Mineo et al., 2006) was able to recapitulate the effect of Bt, with certain exceptions. For example, the regulation of EEC in the colon was weakly affected by APS compared to Bt, which may indicate there is reduced access of oral APS to the colon compared with the small intestine. Alternatively, there may be additional factors and metabolites to APS that convey the efficacy of Bt in the colon. The inability of Lr, which produces a similar profile of SCFA (including acetate and propionate but not succinate) to Bt (Kahouli et al., 2015) to replicate the effects of Bt on EEC supports this proposal. Surprisingly, in several cases, the effects of APS were greater in the colon after a 10-day washout period. This may be indicative of the effects of APS being gradual in onset, and/or their initial effect persisting and being amplified. A long-lasting effect could also arise as a consequence of influencing epithelial stem cells and driving production of EEC lineage cells as recently demonstrated in a SCFA (acetate, propionate, butyrate)-murine and human intestinal enteroid co-culture model system (Pearce et al., 2020). In this *in vitro* culture system, butyrate was shown to be the most effective SCFA in increasing ChrA expression (Pearce et al., 2020). This may explain the loss of ChrA^+^ cells we observed *in vivo* after washout of the butyrate-deficient APS cocktail. The absence of a long lasting effect of APS on ChrA cells might also reflect a separate population of EEC not otherwise labeled in our study that accounted for its transient effect, such as PYY containing L-cells (Aktar et al., 2020) or tuft cells (Sutherland et al., 2007).

Our finding that epithelial cell monolayers generated from small intestinal crypts of GF mice do not accurately reflect the EEC makeup or response to Bt (with the exception of GIP^+^ cells) and APS seen *in vivo* is at odds with their increasing use as a physiologic model of intestinal response to stimuli including microbes and nutrients (Leushacke and Barker, 2014; Pearce et al., 2018; Yin et al., 2019). Our contradictory findings may relate to our use of GF mice, as SPF mice are the usual source of intestinal crypts (Petersen et al., 2014; Roberts et al., 2019). However, the finding that the transcriptome and proteome of small intestinal stem cell-derived organoids from SPF and GF mice co-cluster (Hausmann et al., 2020), would argue against this possibility although in this study no account was made for any possible differences in EEC distribution or number. Indeed, whereas our analysis relied on cellular comparisons of EEC in epithelial cell monolayer cultures versus in intact tissue, other studies have used single or multi-omics-based approaches in comparative studies (Lindeboom et al., 2018; Beumer et al., 2020; Hausmann et al., 2020; Ohki et al., 2020). Other possible confounding factors include comparing EECs in two-dimensional epithelial cell monolayers versus stem cell-derived three-dimensional organoids, other methodological differences including the age of the mice used, where in the small intestine crypts are obtained from Fuller et al. (2012), the duration of culture, and the type and concentrations of growth and differentiation factors used. Additional multidisciplinary studies incorporating both molecular and cellular methodologies are required in order to address these discrepancies, and to determine what aspects of EEC physiology can, and can’t, be faithfully represented by crypt-derived epithelial cell monolayers from conventional versus GF mice.

## Conclusion

We have used conventional and GF mice to demonstrate that the intestinal microbiota is required for regulation of EEC networks, and that a single microbe, Bt, can recapitulate its role in a process that may be dependent on their metabolism and production of APS. Since Bt is a major human symbiont, these findings have implications for novel interventions for the maintenance of human health via the microbiome.

## Data Availability Statement

The original contributions presented in the study are included in the article/Supplementary Material, further inquiries can be directed to the corresponding author.

## Ethics Statement

All experiments were conducted in accordance with the Home Office Animals (Scientific Procedures) Act 1986 under the license number PPL80/2545 at the UEA.

## Author Contributions

SC, AP, and LA conceived and designed the experiments. AM, LA, and SC wrote the manuscript and SC supervised the research. SC, AM, RS, EJ, AG, and AP executed the experimental work. MD undertook the chemometric and metabolite analysis. SC, AM, AP, and LA carried out the data interpretation. AM carried out the statistical analysis. All authors revised, read and approved the final manuscript.

## Conflict of Interest

The authors declare that the research was conducted in the absence of any commercial or financial relationships that could be construed as a potential conflict of interest.

## Abbreviations

APS: acetate propionate succinate
Bt: *Bacteroides thetaiotaomicron*
ChrA: chromogranin A
EC: enterochromaffin cells
EEC: enteroendocrine cells
GIP: gastric inhibitory peptide
GF: germfree
GIT: gastrointestinal tract
GLP-1: glucagon-like peptide-1
5-HT: -hydroxytryptamine
Lr: *Lactobacillus reuteri*
SCFA: short chain fatty acids
SPF: specific pathogen free.
