# Supplementary material for: Regulation of Enteroendocrine Cell Networks by the Major Human Gut Symbiont Bacteroides thetaiotaomicron
Source: Front Microbiol. 2020 Nov 6;11:575595. doi: 10.3389/fmicb.2020.575595 (PMC7677362; doi:10.3389/fmicb.2020.575595)
Supplement: Supplementary file 1 [file Presentation_1.PPTX]

## Slide 1
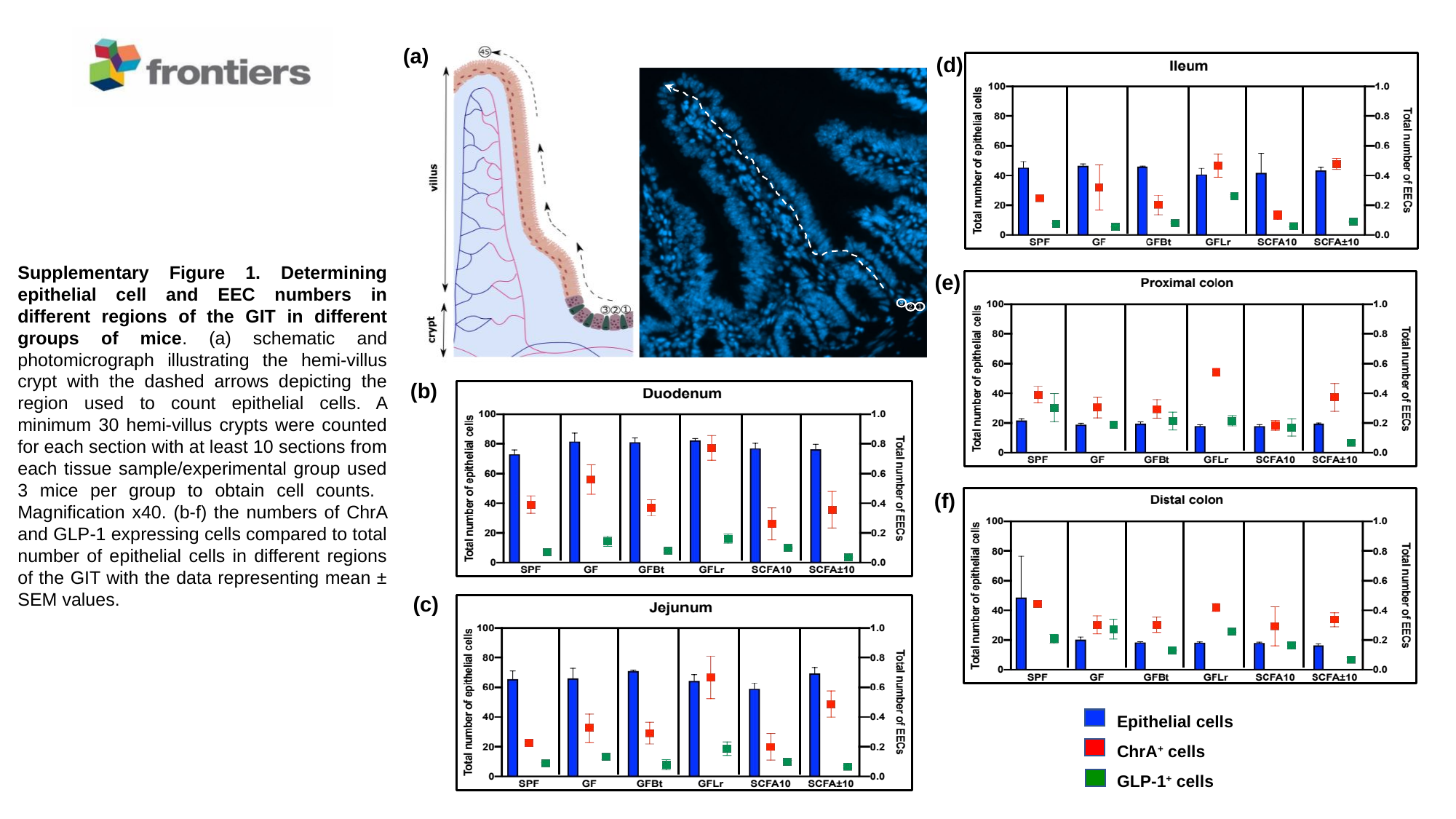

(a)
3
1
2
(d)
Supplementary Figure 1. Determining epithelial cell and EEC numbers in different regions of the GIT in different groups of mice. (a) schematic and photomicrograph illustrating the hemi-villus crypt with the dashed arrows depicting the region used to count epithelial cells. A minimum 30 hemi-villus crypts were counted for each section with at least 10 sections from each tissue sample/experimental group used 3 mice per group to obtain cell counts. Magnification x40. (b-f) the numbers of ChrA and GLP-1 expressing cells compared to total number of epithelial cells in different regions of the GIT with the data representing mean ± SEM values.
(e)
(b)
(f)
(c)
Epithelial cells
ChrA+ cells
GLP-1+ cells

## Slide 2
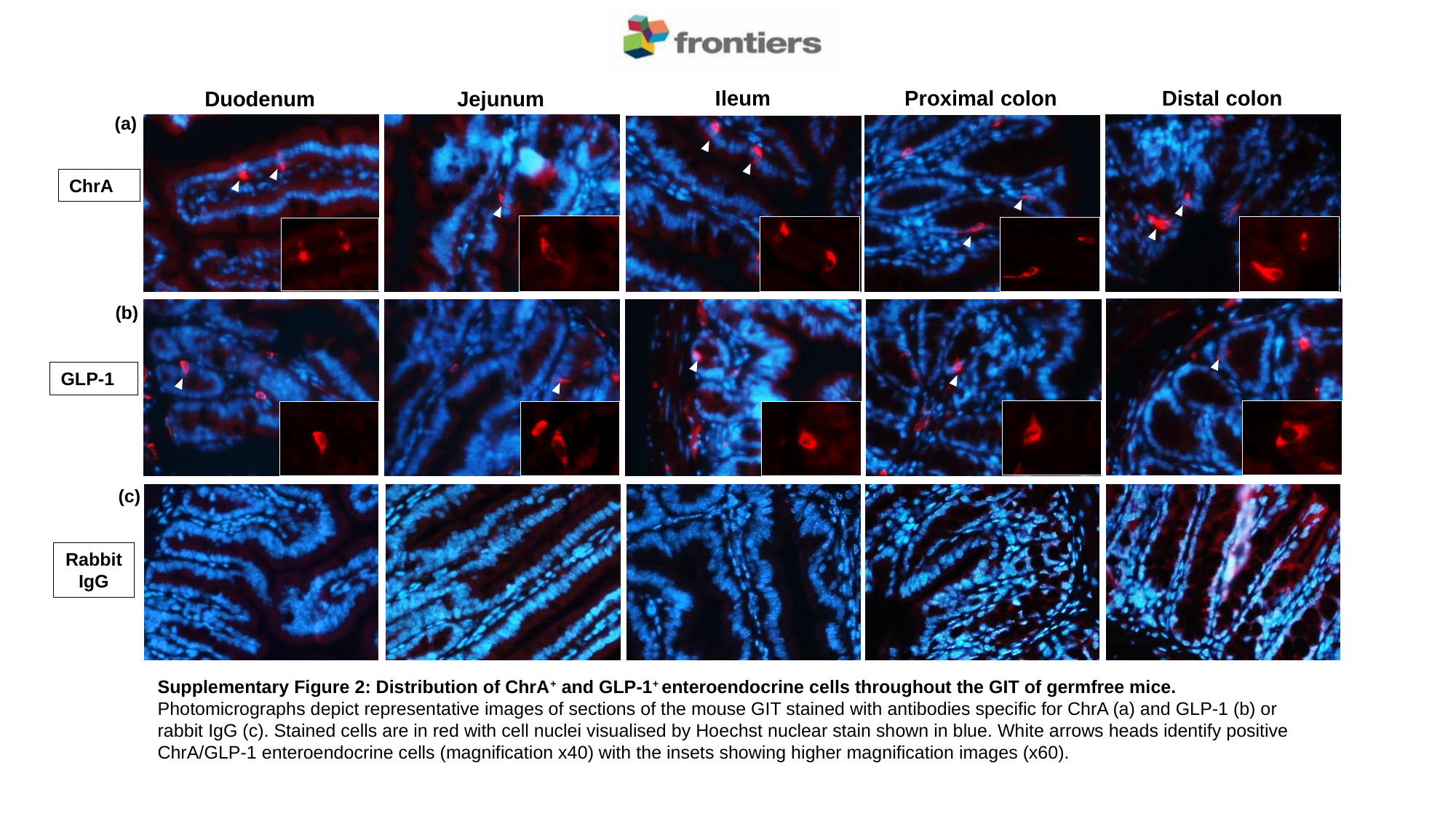

Proximal colon
Ileum
Distal colon
Jejunum
Duodenum
(a)
ChrA
(b)
GLP-1
(c)
Rabbit IgG
Supplementary Figure 2: Distribution of ChrA+ and GLP-1+ enteroendocrine cells throughout the GIT of germfree mice. Photomicrographs depict representative images of sections of the mouse GIT stained with antibodies specific for ChrA (a) and GLP-1 (b) or rabbit IgG (c). Stained cells are in red with cell nuclei visualised by Hoechst nuclear stain shown in blue. White arrows heads identify positive ChrA/GLP-1 enteroendocrine cells (magnification x40) with the insets showing higher magnification images (x60).

## Slide 3
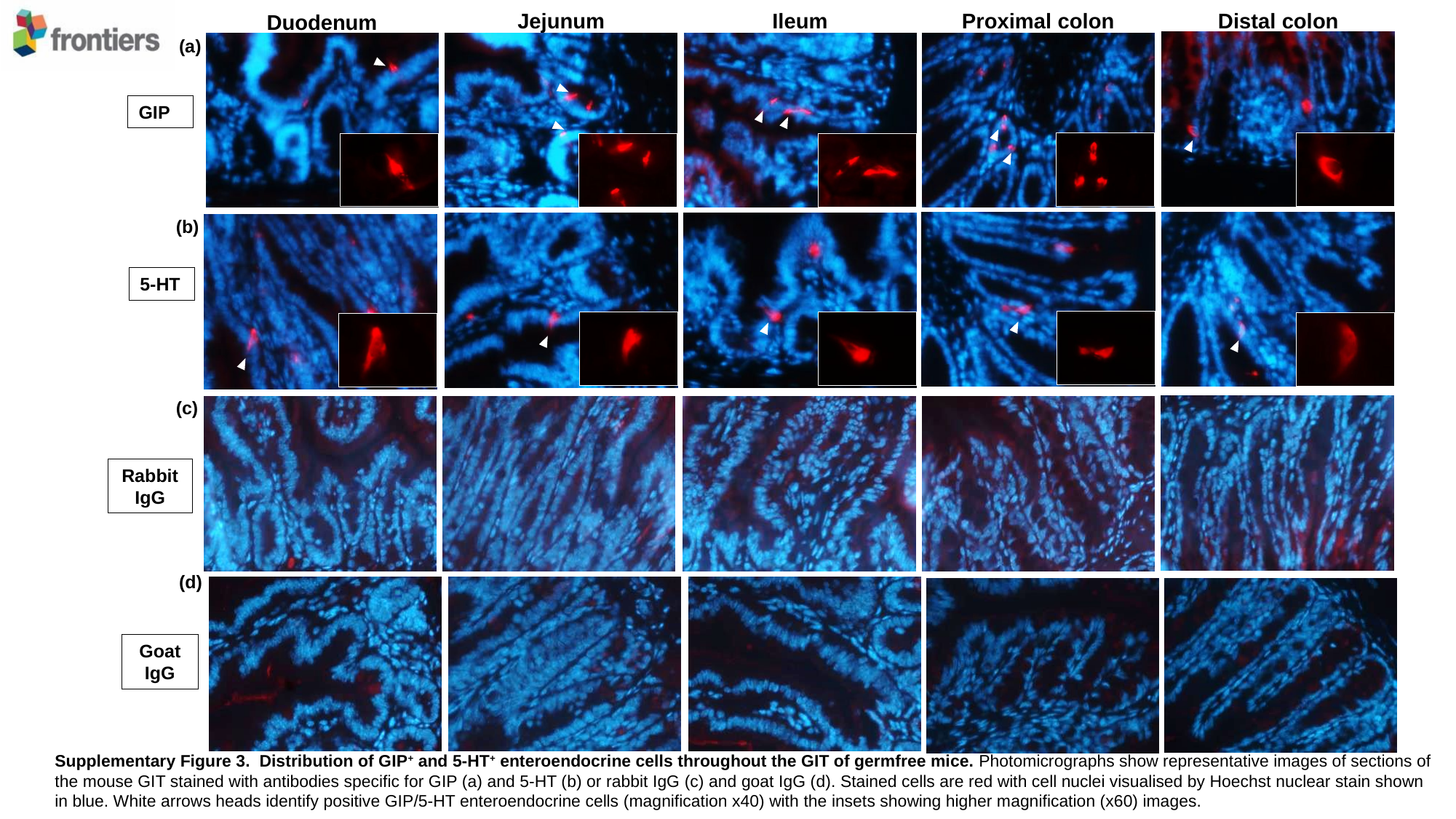

Proximal colon
Distal colon
Ileum
Jejunum
Duodenum
(a)
(b)
(c)
(d)
GIP
5-HT
Rabbit IgG
Goat IgG
Supplementary Figure 3. Distribution of GIP+ and 5-HT+ enteroendocrine cells throughout the GIT of germfree mice. Photomicrographs show representative images of sections of the mouse GIT stained with antibodies specific for GIP (a) and 5-HT (b) or rabbit IgG (c) and goat IgG (d). Stained cells are red with cell nuclei visualised by Hoechst nuclear stain shown in blue. White arrows heads identify positive GIP/5-HT enteroendocrine cells (magnification x40) with the insets showing higher magnification (x60) images.

## Slide 4
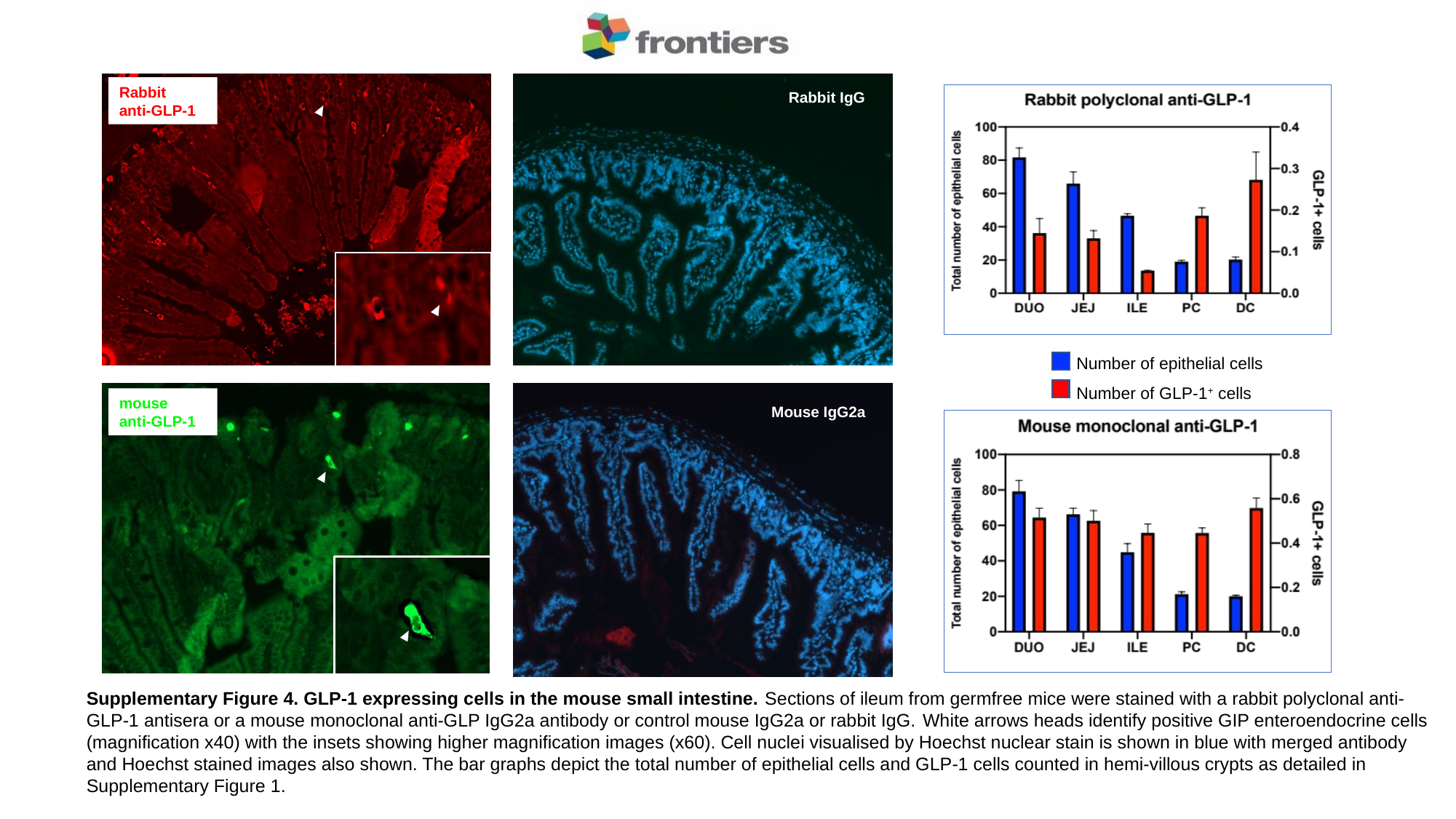

Rabbit
anti-GLP-1
merge
mouse
anti-GLP-1
merge
Rabbit IgG
Number of epithelial cells
Number of GLP-1+ cells
Mouse IgG2a
Supplementary Figure 4. GLP-1 expressing cells in the mouse small intestine. Sections of ileum from germfree mice were stained with a rabbit polyclonal anti-GLP-1 antisera or a mouse monoclonal anti-GLP IgG2a antibody or control mouse IgG2a or rabbit IgG. White arrows heads identify positive GIP enteroendocrine cells (magnification x40) with the insets showing higher magnification images (x60). Cell nuclei visualised by Hoechst nuclear stain is shown in blue with merged antibody and Hoechst stained images also shown. The bar graphs depict the total number of epithelial cells and GLP-1 cells counted in hemi-villous crypts as detailed in Supplementary Figure 1.

## Slide 5
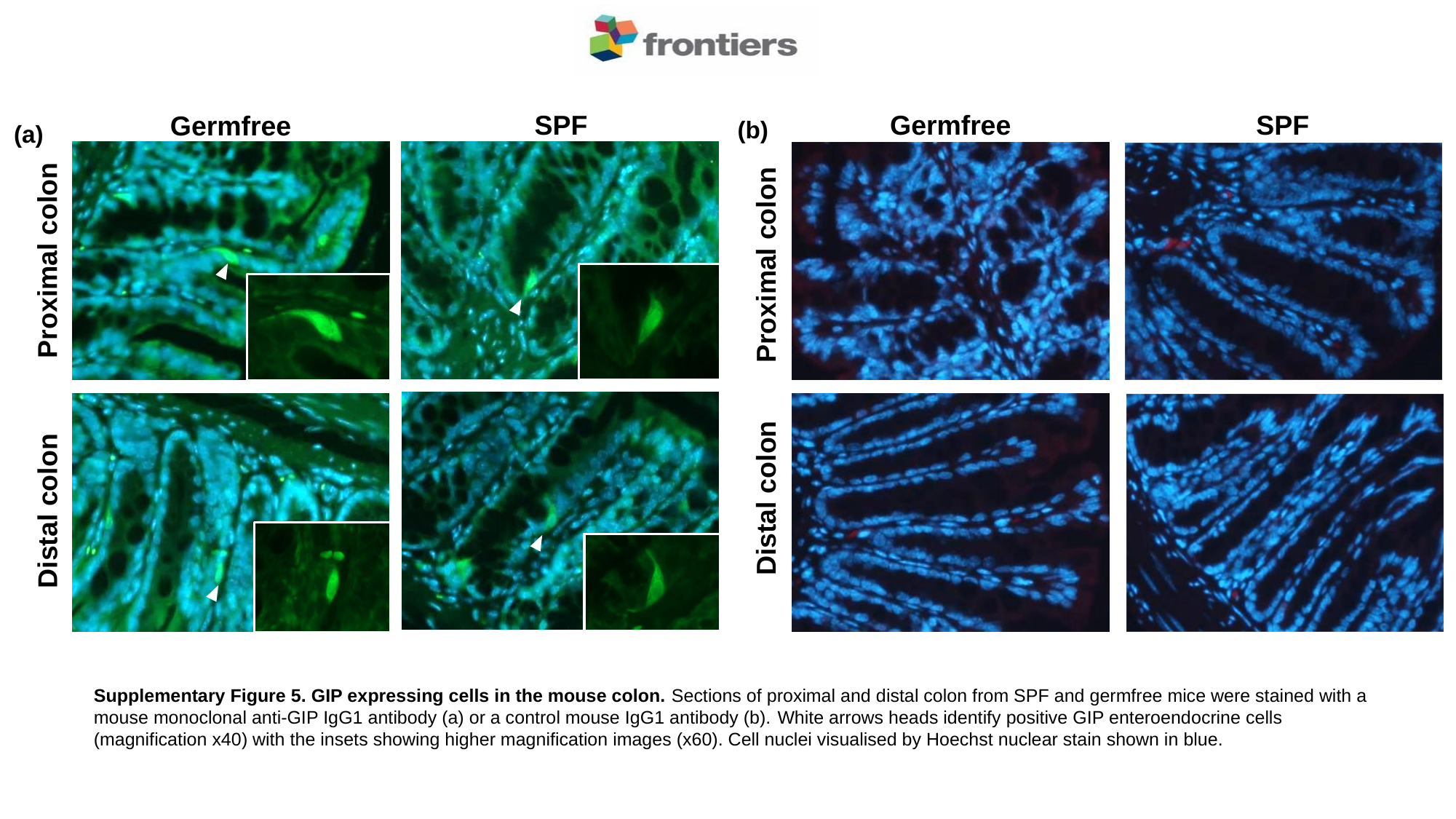

SPF
Germfree
Proximal colon
Distal colon
(a)
Germfree
(b)
Proximal colon
Distal colon
SPF
Supplementary Figure 5. GIP expressing cells in the mouse colon. Sections of proximal and distal colon from SPF and germfree mice were stained with a mouse monoclonal anti-GIP IgG1 antibody (a) or a control mouse IgG1 antibody (b). White arrows heads identify positive GIP enteroendocrine cells (magnification x40) with the insets showing higher magnification images (x60). Cell nuclei visualised by Hoechst nuclear stain shown in blue.

## Slide 6
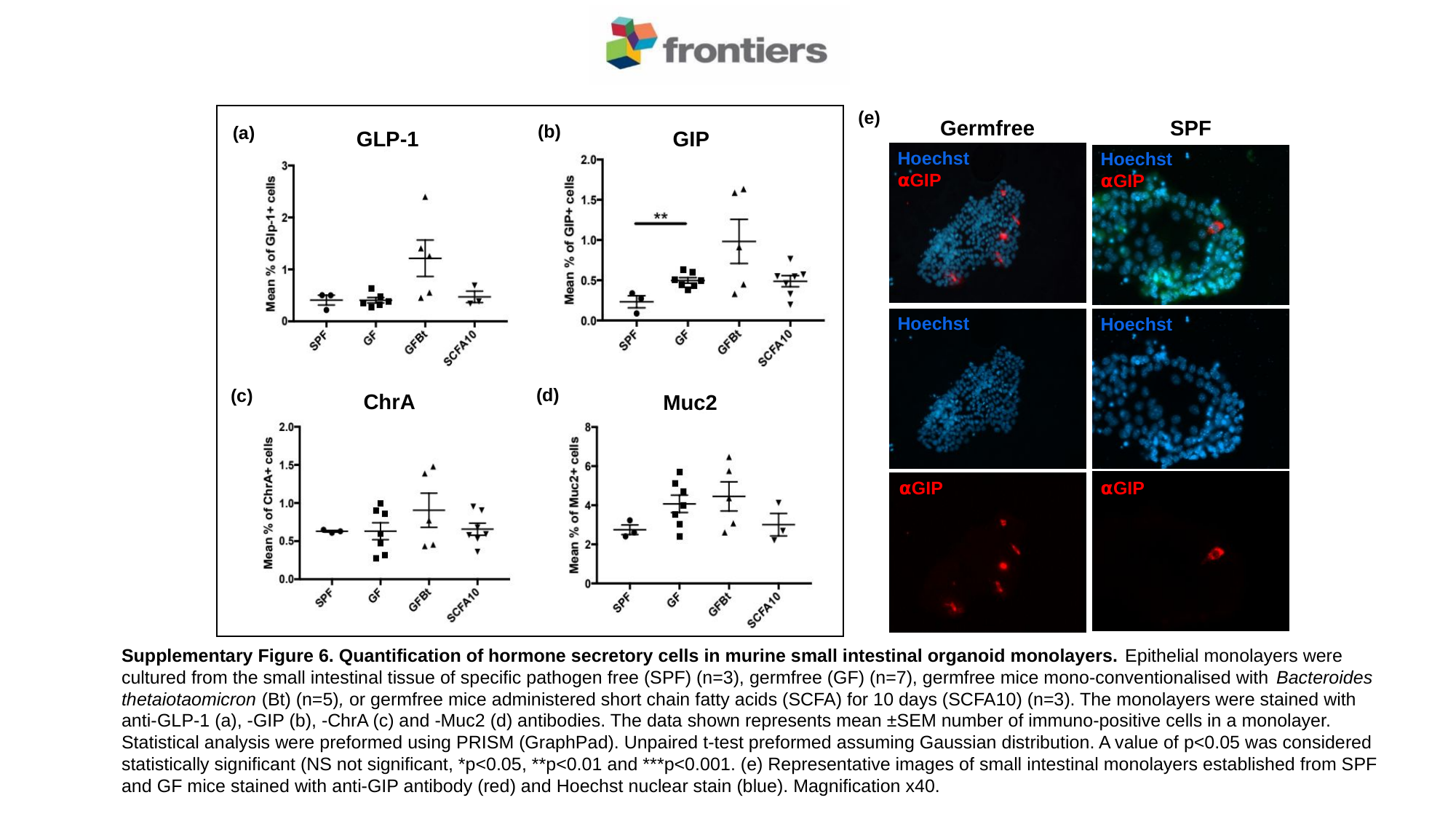

(e)
Germfree
Hoechst
⍺GIP
SPF
(b)
(a)
GLP-1
GIP
(d)
(c)
ChrA
Muc2
Hoechst
⍺GIP
Hoechst
Hoechst
⍺GIP
⍺GIP
Supplementary Figure 6. Quantification of hormone secretory cells in murine small intestinal organoid monolayers. Epithelial monolayers were cultured from the small intestinal tissue of specific pathogen free (SPF) (n=3), germfree (GF) (n=7), germfree mice mono-conventionalised with Bacteroides thetaiotaomicron (Bt) (n=5), or germfree mice administered short chain fatty acids (SCFA) for 10 days (SCFA10) (n=3). The monolayers were stained with anti-GLP-1 (a), -GIP (b), -ChrA (c) and -Muc2 (d) antibodies. The data shown represents mean ±SEM number of immuno-positive cells in a monolayer. Statistical analysis were preformed using PRISM (GraphPad). Unpaired t-test preformed assuming Gaussian distribution. A value of p<0.05 was considered statistically significant (NS not significant, *p<0.05, **p<0.01 and ***p<0.001. (e) Representative images of small intestinal monolayers established from SPF and GF mice stained with anti-GIP antibody (red) and Hoechst nuclear stain (blue). Magnification x40.
