# Supplementary material for: Regulation of Enteroendocrine Cell Networks by the Major Human Gut Symbiont Bacteroides thetaiotaomicron
Source: Front Microbiol. 2020 Nov 6;11:575595. doi: 10.3389/fmicb.2020.575595 (PMC7677362; doi:10.3389/fmicb.2020.575595)
Supplement: Supplementary file 2 [file Presentation_2.PPTX]

## Slide 1
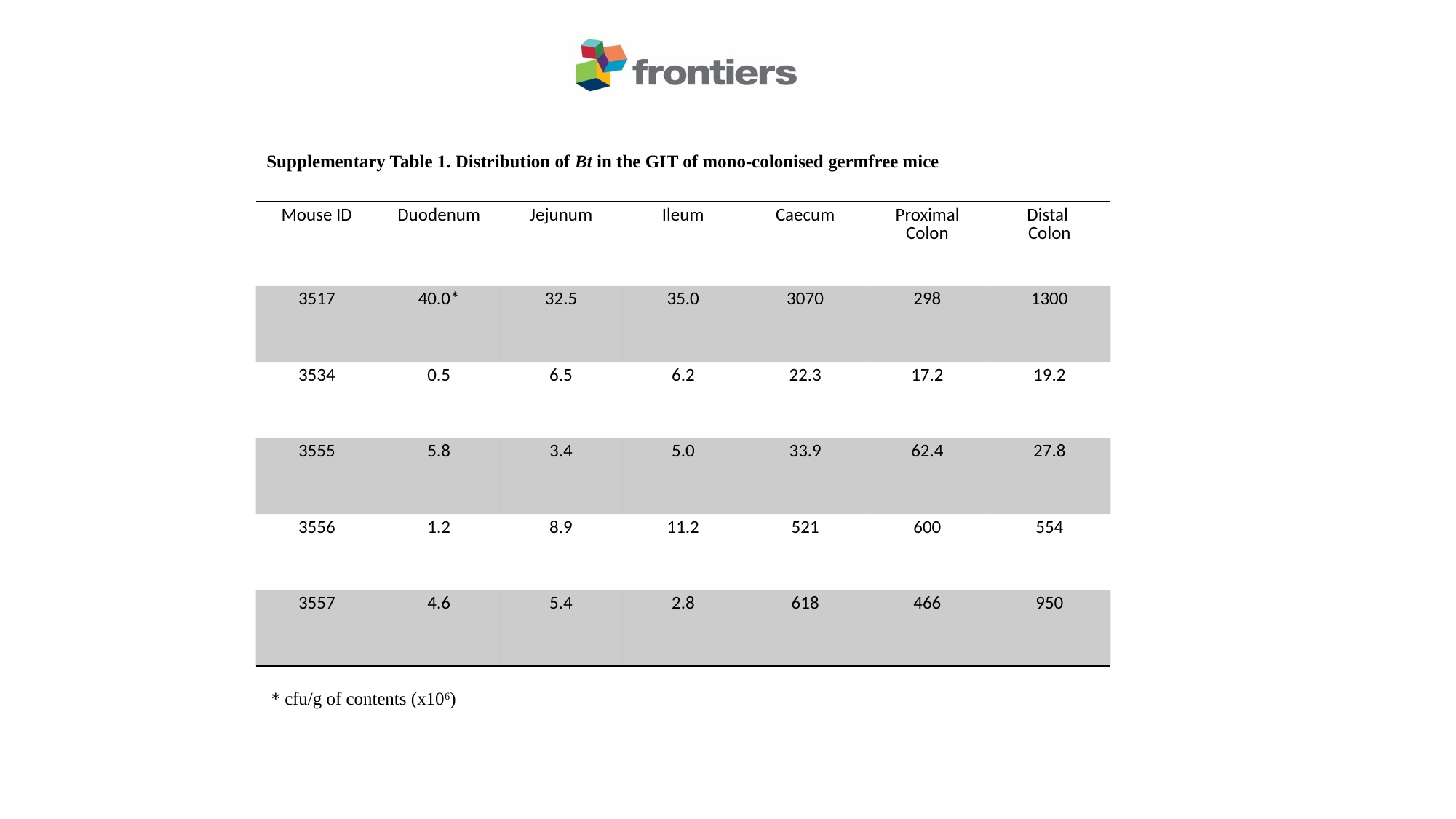

Supplementary Table 1. Distribution of Bt in the GIT of mono-colonised germfree mice
| Mouse ID | Duodenum | Jejunum | Ileum | Caecum | Proximal Colon | Distal Colon |
| --- | --- | --- | --- | --- | --- | --- |
| 3517 | 40.0\* | 32.5 | 35.0 | 3070 | 298 | 1300 |
| 3534 | 0.5 | 6.5 | 6.2 | 22.3 | 17.2 | 19.2 |
| 3555 | 5.8 | 3.4 | 5.0 | 33.9 | 62.4 | 27.8 |
| 3556 | 1.2 | 8.9 | 11.2 | 521 | 600 | 554 |
| 3557 | 4.6 | 5.4 | 2.8 | 618 | 466 | 950 |
 * cfu/g of contents (x106)

## Slide 2
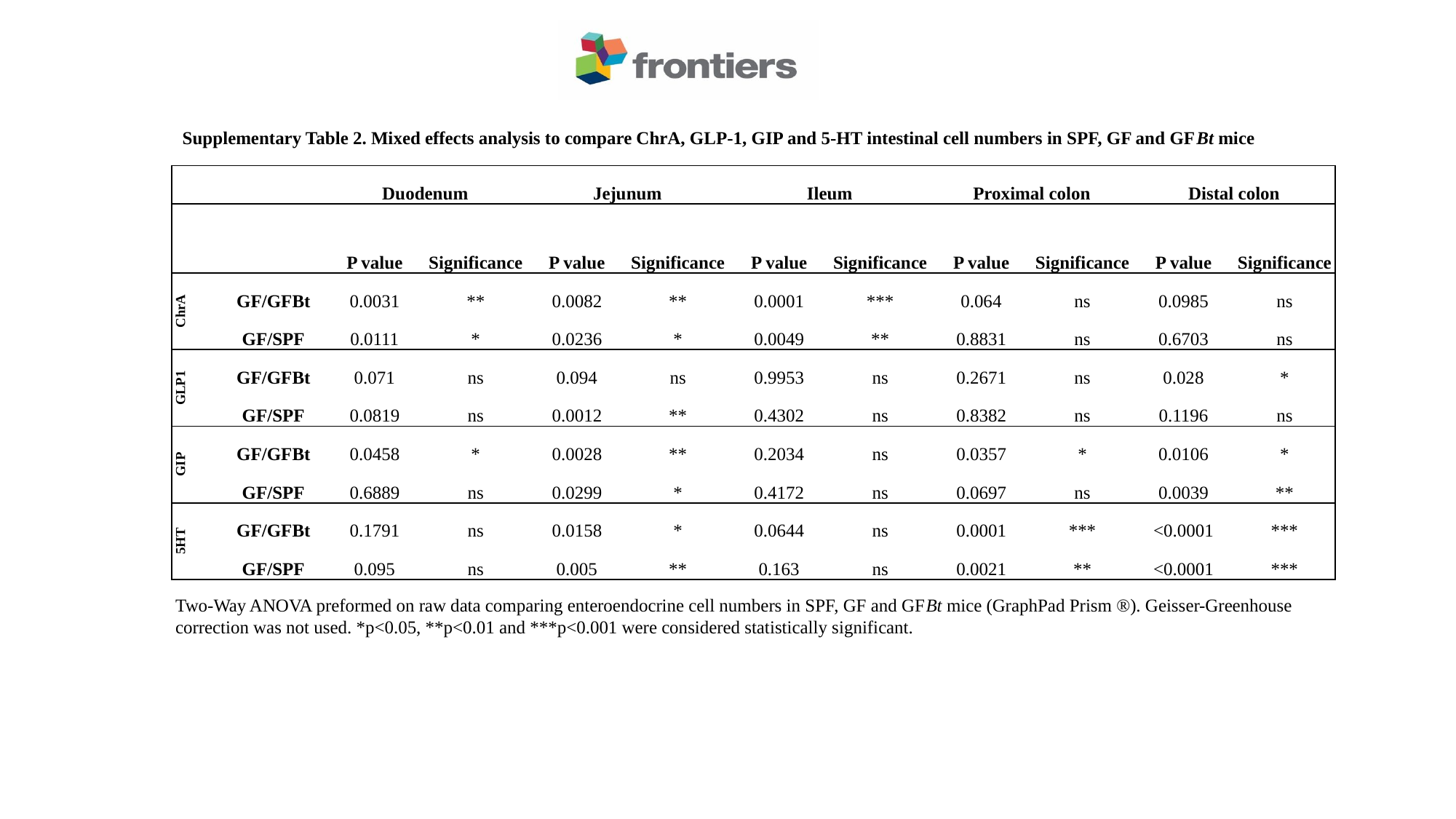

Supplementary Table 2. Mixed effects analysis to compare ChrA, GLP-1, GIP and 5-HT intestinal cell numbers in SPF, GF and GFBt mice
| | | Duodenum | | Jejunum | | Ileum | | Proximal colon | | Distal colon | |
| --- | --- | --- | --- | --- | --- | --- | --- | --- | --- | --- | --- |
| | | P value | Significance | P value | Significance | P value | Significance | P value | Significance | P value | Significance |
| ChrA | GF/GFBt | 0.0031 | \*\* | 0.0082 | \*\* | 0.0001 | \*\*\* | 0.064 | ns | 0.0985 | ns |
| | GF/SPF | 0.0111 | \* | 0.0236 | \* | 0.0049 | \*\* | 0.8831 | ns | 0.6703 | ns |
| GLP1 | GF/GFBt | 0.071 | ns | 0.094 | ns | 0.9953 | ns | 0.2671 | ns | 0.028 | \* |
| | GF/SPF | 0.0819 | ns | 0.0012 | \*\* | 0.4302 | ns | 0.8382 | ns | 0.1196 | ns |
| GIP | GF/GFBt | 0.0458 | \* | 0.0028 | \*\* | 0.2034 | ns | 0.0357 | \* | 0.0106 | \* |
| | GF/SPF | 0.6889 | ns | 0.0299 | \* | 0.4172 | ns | 0.0697 | ns | 0.0039 | \*\* |
| 5HT | GF/GFBt | 0.1791 | ns | 0.0158 | \* | 0.0644 | ns | 0.0001 | \*\*\* | <0.0001 | \*\*\* |
| | GF/SPF | 0.095 | ns | 0.005 | \*\* | 0.163 | ns | 0.0021 | \*\* | <0.0001 | \*\*\* |
Two-Way ANOVA preformed on raw data comparing enteroendocrine cell numbers in SPF, GF and GFBt mice (GraphPad Prism ®). Geisser-Greenhouse correction was not used. *p<0.05, **p<0.01 and ***p<0.001 were considered statistically significant.

## Slide 3
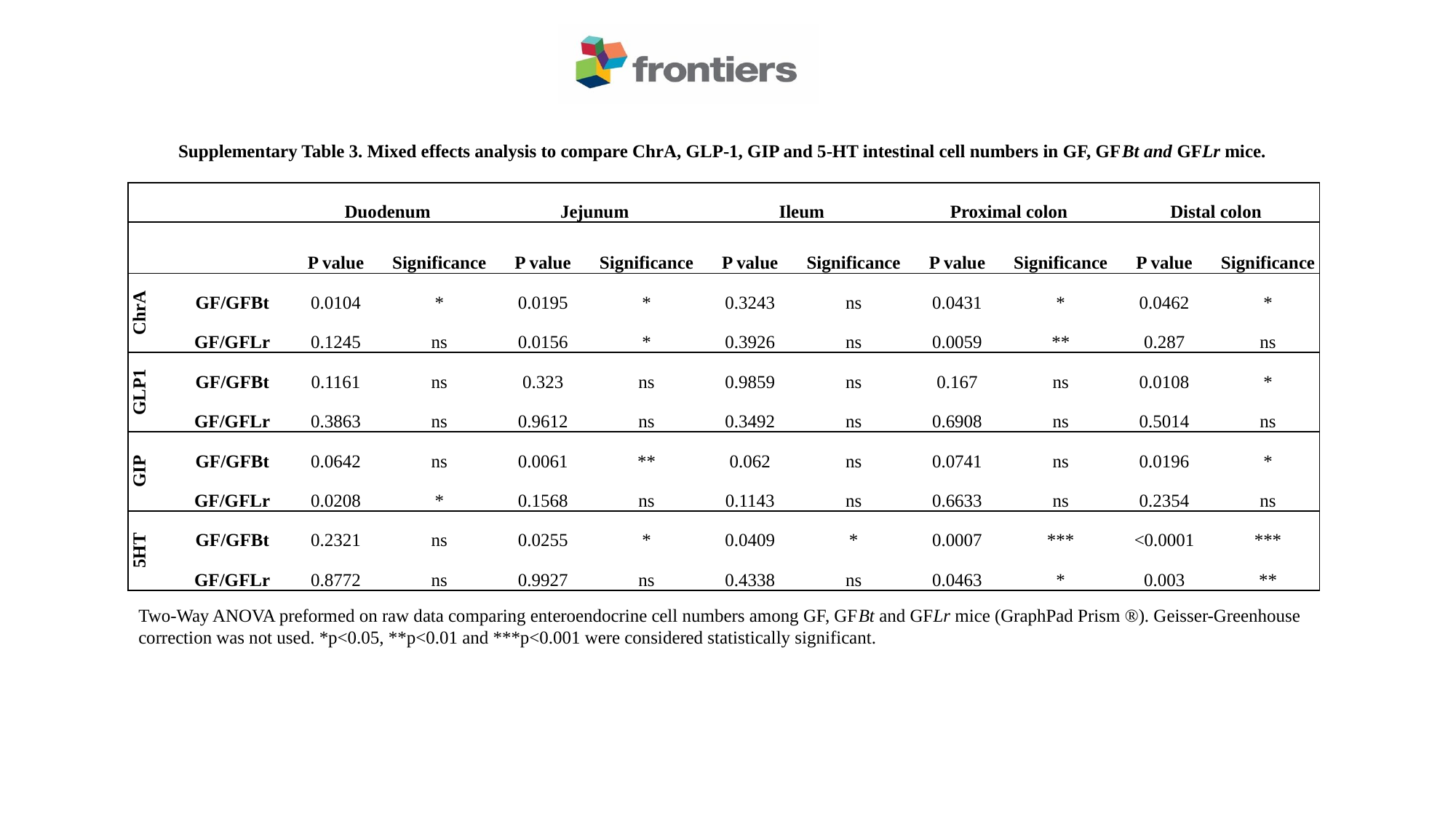

Supplementary Table 3. Mixed effects analysis to compare ChrA, GLP-1, GIP and 5-HT intestinal cell numbers in GF, GFBt and GFLr mice.
| | | Duodenum | | Jejunum | | Ileum | | Proximal colon | | Distal colon | |
| --- | --- | --- | --- | --- | --- | --- | --- | --- | --- | --- | --- |
| | | P value | Significance | P value | Significance | P value | Significance | P value | Significance | P value | Significance |
| ChrA | GF/GFBt | 0.0104 | \* | 0.0195 | \* | 0.3243 | ns | 0.0431 | \* | 0.0462 | \* |
| | GF/GFLr | 0.1245 | ns | 0.0156 | \* | 0.3926 | ns | 0.0059 | \*\* | 0.287 | ns |
| GLP1 | GF/GFBt | 0.1161 | ns | 0.323 | ns | 0.9859 | ns | 0.167 | ns | 0.0108 | \* |
| | GF/GFLr | 0.3863 | ns | 0.9612 | ns | 0.3492 | ns | 0.6908 | ns | 0.5014 | ns |
| GIP | GF/GFBt | 0.0642 | ns | 0.0061 | \*\* | 0.062 | ns | 0.0741 | ns | 0.0196 | \* |
| | GF/GFLr | 0.0208 | \* | 0.1568 | ns | 0.1143 | ns | 0.6633 | ns | 0.2354 | ns |
| 5HT | GF/GFBt | 0.2321 | ns | 0.0255 | \* | 0.0409 | \* | 0.0007 | \*\*\* | <0.0001 | \*\*\* |
| | GF/GFLr | 0.8772 | ns | 0.9927 | ns | 0.4338 | ns | 0.0463 | \* | 0.003 | \*\* |
Two-Way ANOVA preformed on raw data comparing enteroendocrine cell numbers among GF, GFBt and GFLr mice (GraphPad Prism ®). Geisser-Greenhouse correction was not used. *p<0.05, **p<0.01 and ***p<0.001 were considered statistically significant.

## Slide 4
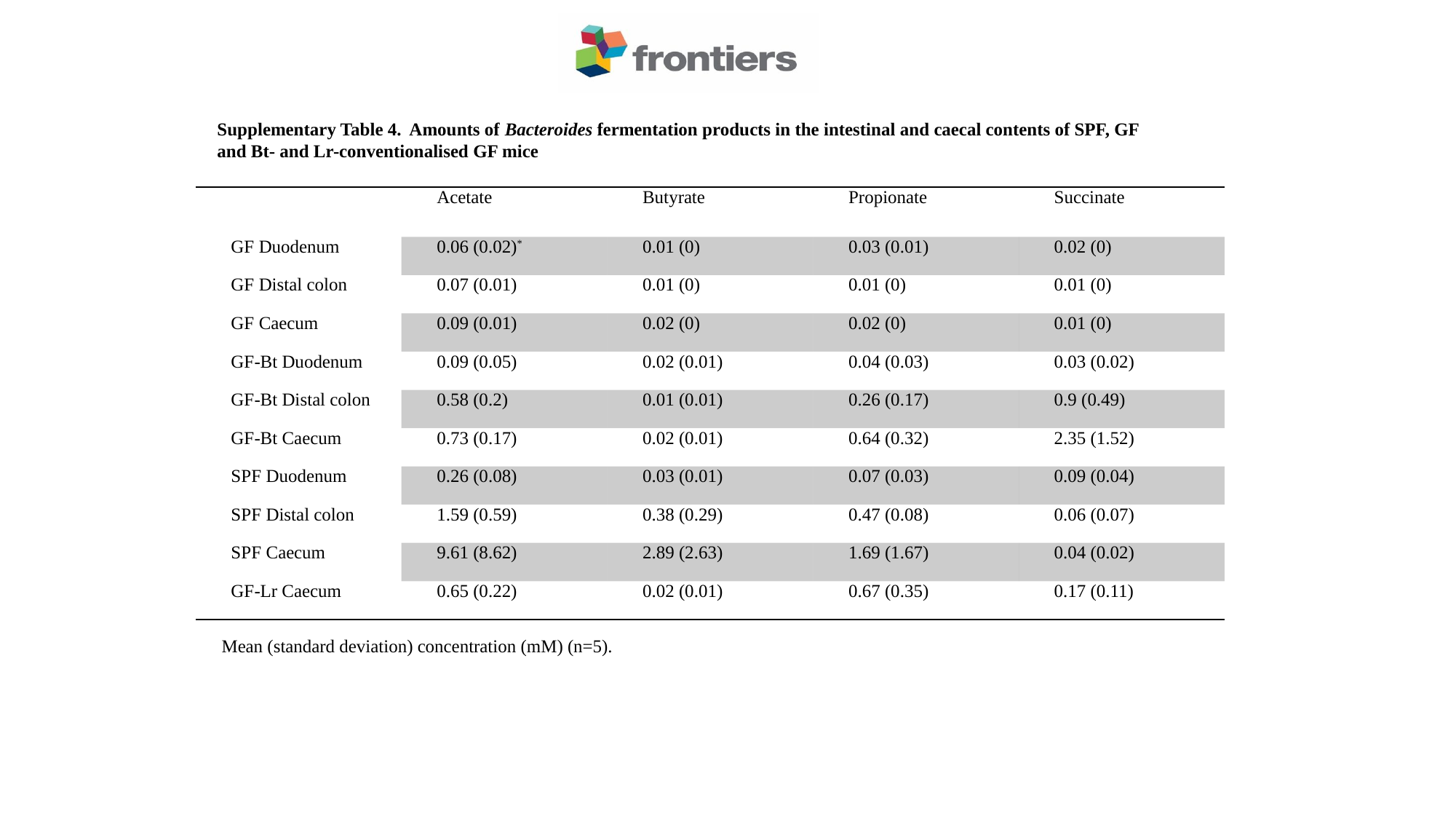

Supplementary Table 4. Amounts of Bacteroides fermentation products in the intestinal and caecal contents of SPF, GF and Bt- and Lr-conventionalised GF mice
| | Acetate | Butyrate | Propionate | Succinate |
| --- | --- | --- | --- | --- |
| GF Duodenum | 0.06 (0.02)\* | 0.01 (0) | 0.03 (0.01) | 0.02 (0) |
| GF Distal colon | 0.07 (0.01) | 0.01 (0) | 0.01 (0) | 0.01 (0) |
| GF Caecum | 0.09 (0.01) | 0.02 (0) | 0.02 (0) | 0.01 (0) |
| GF-Bt Duodenum | 0.09 (0.05) | 0.02 (0.01) | 0.04 (0.03) | 0.03 (0.02) |
| GF-Bt Distal colon | 0.58 (0.2) | 0.01 (0.01) | 0.26 (0.17) | 0.9 (0.49) |
| GF-Bt Caecum | 0.73 (0.17) | 0.02 (0.01) | 0.64 (0.32) | 2.35 (1.52) |
| SPF Duodenum | 0.26 (0.08) | 0.03 (0.01) | 0.07 (0.03) | 0.09 (0.04) |
| SPF Distal colon | 1.59 (0.59) | 0.38 (0.29) | 0.47 (0.08) | 0.06 (0.07) |
| SPF Caecum | 9.61 (8.62) | 2.89 (2.63) | 1.69 (1.67) | 0.04 (0.02) |
| GF-Lr Caecum | 0.65 (0.22) | 0.02 (0.01) | 0.67 (0.35) | 0.17 (0.11) |
Mean (standard deviation) concentration (mM) (n=5).

## Slide 5
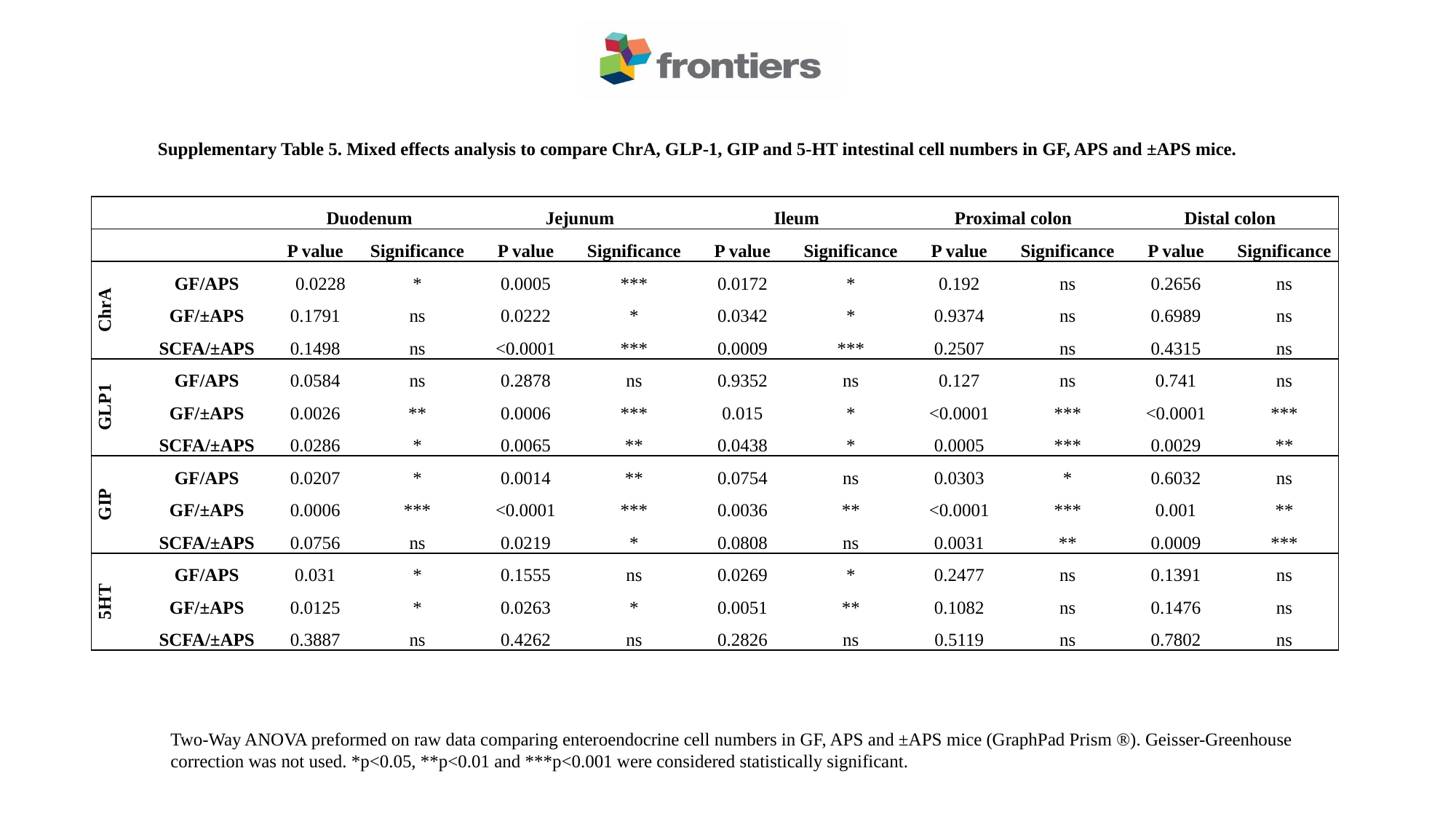

Supplementary Table 5. Mixed effects analysis to compare ChrA, GLP-1, GIP and 5-HT intestinal cell numbers in GF, APS and ±APS mice.
| | | Duodenum | | Jejunum | | Ileum | | Proximal colon | | Distal colon | |
| --- | --- | --- | --- | --- | --- | --- | --- | --- | --- | --- | --- |
| | | P value | Significance | P value | Significance | P value | Significance | P value | Significance | P value | Significance |
| ChrA | GF/APS | 0.0228 | \* | 0.0005 | \*\*\* | 0.0172 | \* | 0.192 | ns | 0.2656 | ns |
| | GF/±APS | 0.1791 | ns | 0.0222 | \* | 0.0342 | \* | 0.9374 | ns | 0.6989 | ns |
| | SCFA/±APS | 0.1498 | ns | <0.0001 | \*\*\* | 0.0009 | \*\*\* | 0.2507 | ns | 0.4315 | ns |
| GLP1 | GF/APS | 0.0584 | ns | 0.2878 | ns | 0.9352 | ns | 0.127 | ns | 0.741 | ns |
| | GF/±APS | 0.0026 | \*\* | 0.0006 | \*\*\* | 0.015 | \* | <0.0001 | \*\*\* | <0.0001 | \*\*\* |
| | SCFA/±APS | 0.0286 | \* | 0.0065 | \*\* | 0.0438 | \* | 0.0005 | \*\*\* | 0.0029 | \*\* |
| GIP | GF/APS | 0.0207 | \* | 0.0014 | \*\* | 0.0754 | ns | 0.0303 | \* | 0.6032 | ns |
| | GF/±APS | 0.0006 | \*\*\* | <0.0001 | \*\*\* | 0.0036 | \*\* | <0.0001 | \*\*\* | 0.001 | \*\* |
| | SCFA/±APS | 0.0756 | ns | 0.0219 | \* | 0.0808 | ns | 0.0031 | \*\* | 0.0009 | \*\*\* |
| 5HT | GF/APS | 0.031 | \* | 0.1555 | ns | 0.0269 | \* | 0.2477 | ns | 0.1391 | ns |
| | GF/±APS | 0.0125 | \* | 0.0263 | \* | 0.0051 | \*\* | 0.1082 | ns | 0.1476 | ns |
| | SCFA/±APS | 0.3887 | ns | 0.4262 | ns | 0.2826 | ns | 0.5119 | ns | 0.7802 | ns |
Two-Way ANOVA preformed on raw data comparing enteroendocrine cell numbers in GF, APS and ±APS mice (GraphPad Prism ®). Geisser-Greenhouse correction was not used. *p<0.05, **p<0.01 and ***p<0.001 were considered statistically significant.
